# Supramolecular self-assembly of EGCG–cysteine nanodrugs for ferroptosis and oxidative stress inhibition in chondrocytes to treat osteoarthritis

**DOI:** 10.1016/j.mtbio.2026.102978

**Published:** 2026-03-10

**Authors:** Zhao Lin, Jiayao Zhang, Peng Zhang, Mingjuan Zhang, Hanhao Dai, Yibin Su, Haiqi Ding, Linhai Yang, Guoming Liu, Jie Xu, Jun Luo

**Affiliations:** a Department of Orthopedics, Shengli Clinical Medical College of Fujian Medical University, Fujian Provincial Hospital, Fuzhou University Affiliated Provincial Hospital, Fuzhou, China; b Department of Orthopedics, Fujian Provincial Hospital, Fuzhou University Affiliated Provincial Hospital, School of Medicine, Fuzhou, China; c Department of Traditional Chinese Medicine, Fujian University of Traditional Chinese Medicine, Fuzhou, China; d Fujian Provincial Clinical Research Center for Spinal Nerve and Joint Diseases, Fuzhou University Affiliated Provincial Hospital, Fuzhou, Fujian, China; e Engineering Research Center of Orthopedic Biomaterials, Fujian Province University, Fuzhou University, Fuzhou, Fujian, China

**Keywords:** Osteoarthritis, Nanoparticles, EC NPs, Ferroptosis, Cartilage matrix

## Abstract

Osteoarthritis (OA) is a common chronic degenerative joint disease that is characterized mainly by the destruction of articular cartilage, synovial inflammation and osteophyte formation, and seriously affects the quality of life of middle-aged and elderly individuals. Recent studies have shown that ferroptosis plays an important role in the development of OA. The aim of this study was to utilize nanoparticles (EC NPs) formed by the self-assembly of epigallocatechin-3-gallate (EGCG) and cysteine to treat OA by inhibiting ferroptosis. The properties of the EC NPs were evaluated at the molecular level, and their therapeutic effects on H_2_O_2_-stimulated chondrocytes were verified. At the molecular level, EC NPs inhibited ROS levels, abnormal accumulation of Fe^2+^ and lipid peroxidation, elevated the expression of glutathione peroxidase 4 (GPX4) to inhibit ferroptosis, repaired cartilage metabolism disorders, and alleviated the progression of OA. Transcriptomic analysis further revealed that EC NPs could exert therapeutic effects by inhibiting multiple inflammatory signaling pathways. To verify their efficacy in vivo, the present study used a mouse medial meniscus instability (DMM)-induced OA model, and EC NPs were administered via intra-articular injection. The results showed that EC NPs were able to significantly attenuate damage to the cartilage matrix and delay the pathological progression of OA. In conclusion, the use of EC NPs, as a green, simple and efficient biotherapeutic strategy, is expected to provide new ideas and methods for the clinical treatment of OA.

## Graphical abstract

**Figure:**
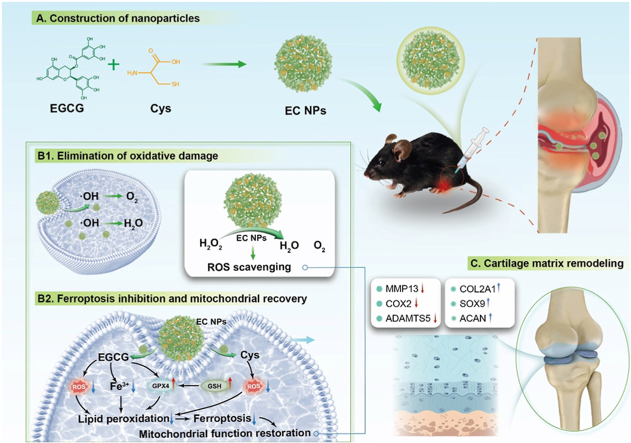

## 1 Introduction

**Scheme 1:**
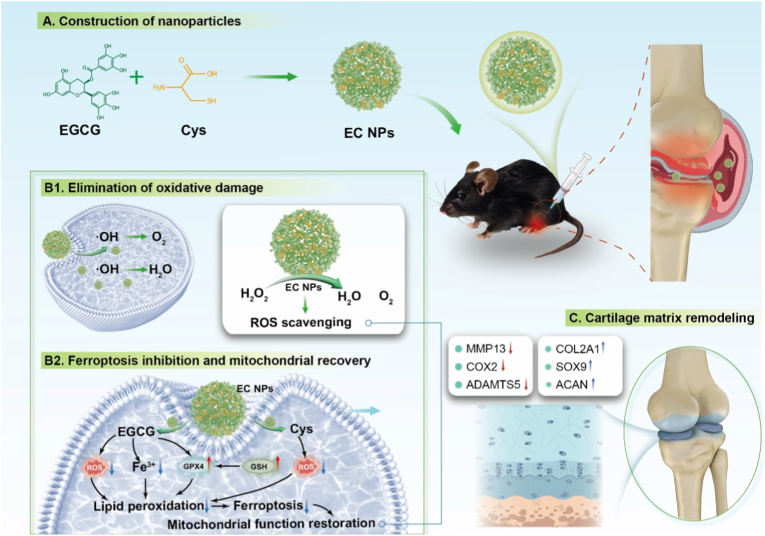
EC NPs were constructed and their effects on chondrocytes were explored, and the results of intra-articular injection for OA were evaluated. (A) The construction process of EC NPs. (B) EC NPs inhibit ferroptosis and oxidative stress in chondrocytes. (C) EC NPs alleviate the progression of OA by inhibiting ferroptosis.

Osteoarthritis (OA) is a common chronic degenerative joint disease characterized by the progressive destruction of joint cartilage, chronic inflammation of synovial tissue, abnormal remodeling of subchondral bone, and the formation of osteophytes [1].OA is an age-related degenerative joint disease with a markedly increased incidence in middle-aged and elderly individuals, substantially impairing quality of life and daily functioning [[2], [3], [4], [5]]. The pathogenesis of OA has not yet been fully elucidated, but studies have shown that a combination of factors, such as abnormal mechanical loading, chronic inflammation, elevated oxidative stress, and disturbances in chondrocyte metabolism, are involved in the occurrence and development of OA [[1], [2], [3], [4], [5], [6], [7], [8], [9]]– (see Scheme 1)

In recent years, a novel mode of cell death, ferroptosis, has gradually attracted the attention of researchers. Ferroptosis is an iron-dependent form of programmed cell death characterized by lipid peroxidation, which is fundamentally different from traditional forms of cell death, such as apoptosis, necrosis and autophagy [10,11]. In the core mechanism of ferroptosis, intracellular free iron ions catalyze lipid peroxidation via the Fenton reaction, leading to disruption of the lipid layer of the cell membrane and ultimately triggering cell death [12,13]. In the pathological process of OA, the homeostasis of intracellular iron ions in chondrocytes is significantly imbalanced, the concentration of free iron ions is significantly elevated, and the expression level of glutathione peroxidase 4 (GPX4) is significantly reduced, resulting in weakening of the intracellular antioxidant system and massive accumulation of lipid peroxides, which further exacerbates the degradation of the cartilaginous extracellular matrix (ECM) and inflammation and thus accelerates the progression of OA [14,15]. Given the critical role of ferroptosis in the pathogenesis of osteoarthritis (OA), the present study proposes a ferroptosis-targeting therapeutic strategy based on nanoparticles (EC NPs) formed by the self-assembly of epigallocatechin-3-gallate (EGCG), a naturally occurring tea polyphenol, with cysteine, to exploit their antioxidant, anti-inflammatory, and chondroprotective properties [16,17]. However, EGCG suffers from poor stability, easy oxidative degradation, and low bioavailability in practical applications, which severely limits its clinical promotion and application. To overcome the above defects of EGCG, cysteine molecules rich in sulfhydryl groups (-SH) were introduced in this study. Cysteine not only has the ability to directly scavenge intracellular reactive oxygen species (ROS) but also serves as an important precursor substance for glutathione (GSH) synthesis, which further enhances the cellular antioxidant defense system [18,19]. Through noncovalent interactions between EGCG and cysteine, including hydrogen bonding, hydrophobic interactions, and π‒π stacking, the two can spontaneously assemble to form stable nanoparticle structures (EC NPs). This self-assembly strategy not only significantly improved the chemical stability and bioavailability of EGCG but also fully exploited the synergistic antioxidant effect between EGCG and cysteine.

On the one hand, the EC NPs were able to effectively scavenge excessive intracellular ROS and reduce the level of lipid peroxidation through multitarget regulation of the ferroptosis pathway, and on the other hand, they restored the function of the intracellular antioxidant system by increasing the expression of GPX4, thus effectively inhibiting the ferroptosis of chondrocytes [14,[20], [21], [22]]. In addition, EC NPs significantly reduce the expression level of inflammatory factors, improve the metabolic disordered state of chondrocytes, and promote the synthesis and repair of the cartilage extracellular matrix (ECM), which ultimately alleviates the progression of OA. Therefore, the EC NPs designed in this study provide a safe, effective and easy-to-implement novel strategy for the clinical treatment of OA and have good application prospects.

## 2 Results

### 2.1 Synthesis and characterization of the EC NPs

**Fig. 1:**
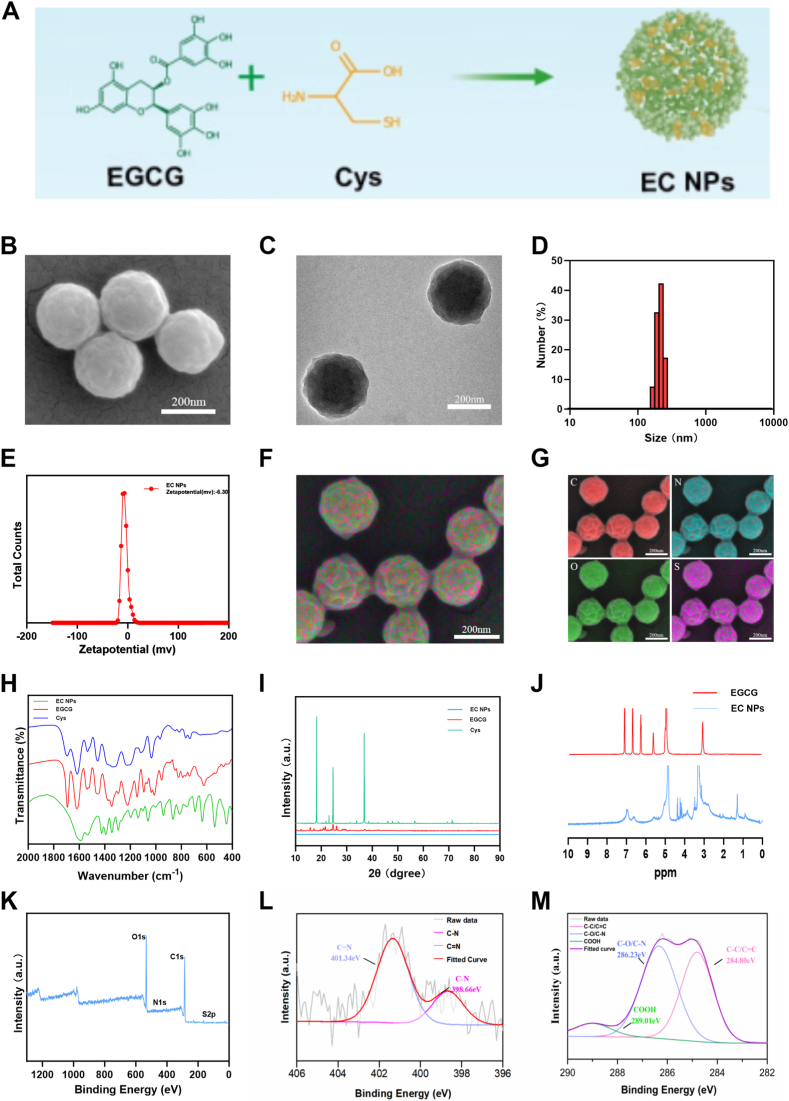
Physicochemical properties of EC NPs. (A) The construction process of EC NPs. (B) SEM images of EC NPs. (C) TEM images of EC NPs. (D) DLS results of EC NPs. (E) Zeta potential results of EC NPs. (F, G) EDS elemental energy spectra of EC NPs. (H) FT-IR spectra of EC NPs, EGCG and Cys. (I) XRD spectra of EC NPs, EGCG and Cys. (J) 1H NMR spectra of EGCG and EC NPs. (K) XPS spectra of EC NPs. (L) N 1s spectra of EC NPs. (M) C 1s spectrum of EC NPs.

Natural polyphenol (EGCG)-Cys nanoparticles were prepared via a one-pot reaction method. Briefly, natural polyphenol (EGCG) was fully dissolved in a mixture of water and methanol at room temperature, and the corresponding mass of Cys was further added to the above mixture. After gentle stirring for 30min, the transparent solution gradually became opaque. The NPs were subsequently collected via centrifugation (10,000 rpm, 10 min) and washed three times with deionized water (Fig. 1) [23]. As shown in Fig. 1B and C, scanning electron microscopy (SEM) and transmission electron microscopy (TEM) observations indicate that the EC nanoparticles are spherical in shape. As shown in Fig. 1D and E, the particle size of the EC NPs was approximately 220.2 nm, with a negative charge of −6.53 mV, while the PDI value was 0.261, indicating that the prepared NPs had a uniform particle size morphology and excellent dispersibility in water. Previous studies have shown that nanoparticles of this size can effectively penetrate the dense matrix of articular cartilage and improve drug delivery and efficacy [24]. To further verify whether EC NPs were synthesized successfully, we subsequently analyzed the elemental composition, valence and surface functional groups of the EC NPs. As shown in Fig. 1F and G, energy dispersive X-ray spectroscopy (EDS) elemental mapping revealed that the elements C, N, O, and S were uniformly distributed in the EC NPs, confirming the successful synthesis of EGCG with Cys and that all of these elements occur naturally in vivo, which reflects the good biocompatibility of the NPs.

As shown in Fig. 1H, Fourier transform infrared spectroscopy (FTIR) revealed that the EC NPs retained the presence of the EGCG molecular backbone, which is consistent with previous studies. As shown in Fig. 1I, X-ray diffraction (XRD) analysis of EC NPs, EGCG, and Cys was performed, and the XRD spectrum of Cys showed distinct diffraction peaks, whereas upon binding with EGCG, the EC NPs presented an amorphous structure. As shown in Fig. 1J, the presence of sulfhydryl groups at 1.4 ppm in the 1H nuclear magnetic resonance (1H NMR) spectra of the EC NPs confirmed the presence of Cys. As shown in the X-ray photoelectron spectra (XPS) in Fig. 1K-M, the peaks at 398.01 eV and 400.31 eV in the N spectrum belong to C─N and C = N, which suggests that the synthesis process of EGCG and Cys forms EGCG-Cys oligomers through Mannich condensation and Schiff base reactions, which are subsequently driven by noncovalent interactions such as hydrogen bonding and π‒π stacking assembly into intact spherical EC NPs [25]. As shown in Fig. S2, the UV‒visible spectrum of the EC NPs has a characteristic absorption peak at approximately 275 nm, which matches the characteristic absorption peak of free EGCG. As shown in Fig. S3, we also performed matrix-assisted laser desorption/ionization time-of-flight (MALDI-TOF) mass spectrometry on the EC NPs to analyze their molecular structures. The mass spectra revealed the presence of many peak clusters at 569.069, 745.455–758.495, 997.28–1008.51, and 1252.573, corresponding to EGCG/Cys monomeric adducts, EGCG with two Cys molecules, EGCG-derived dimers with or without Cys, and trimers with or without Cys, respectively, which allowed us to speculate on the possible molecular formulas of the reaction products between EGCG and Cys. In summary, we successfully synthesized natural polyphenol EGCG-Cys nanoparticles via a one-pot method.

### 2.2 Free radical scavenging capacity, biocompatibility and cellular uptake of the EC NPs

**Fig. 2:**
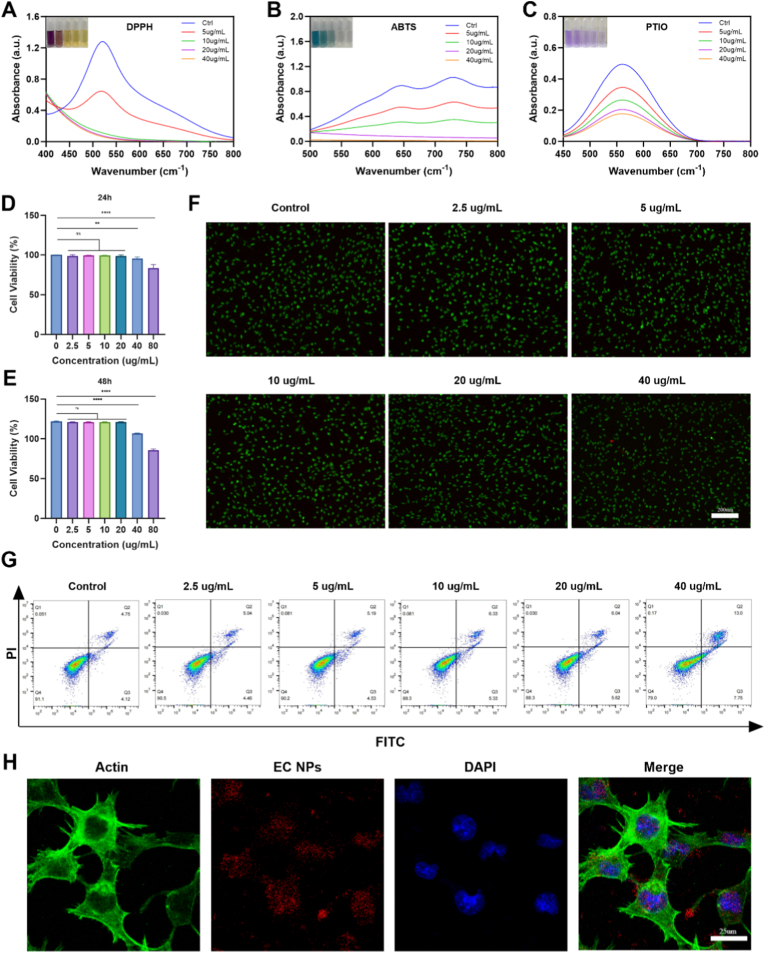
Free radical scavenging, in vitro biocompatibility and cellular uptake of EC NPs. (A-C) Scavenging effects of EC NPs on DPPH, ABTS and PTIO. (D,E) Cell viability of different concentrations of EC NPs co-cultured with chondrocytes for 24 h and 48 h. (F) Live/dead staining images of different concentrations of EC NPs co-cultured with chondrocytes for 24 h. (G) FCM analysis of chondrocyte apoptosis after treatment with different concentrations of EC NPs. (H)Images of chondrocyte uptake of EC NPs. The error bars indicate the mean ± standard deviation. ns (no statistical significance), ∗P < 0.05, ∗∗P < 0.01, ∗∗∗P < 0.001, ∗∗∗∗P < 0.0001.

Osteoarthritis (OA) is closely related to the abnormal accumulation of reactive oxygen species (ROS) in chondrocytes, and EGCG, a natural antioxidant, is able to inhibit the process of osteoarthritis by eliminating the excessive ROS produced by chondrocytes quickly and efficiently because of its strong antioxidant capacity [26,27]. The antioxidant capacity of EC NPs was evaluated via free radical scavenging assays of 1,1-diphenyl-2-picrylhydrazyl (DPPH), 2,2′-azinobis(3-ethylbenzothiazoline-6-sulfonic acid) (ABTS) and 2-phenyl-4,4,5,5-tetramethylimidazoline-1-oxo 3-oxide (PTIO). The scavenging rates of DPPH, ABTS and PTIO radicals increased with increasing concentrations of EC NPs (Fig. 2A–C), indicating that EC NPs have good antioxidant capacity.

Then, the CCK-8 method was used to evaluate the effect of EC NPs on chondrocyte viability. As shown in Fig. 2D–E, after co-culturing chondrocytes with EC NPs for 24 h and 48 h, the concentration ranged from 0 to 20 μg/mL and exhibited good biocompatibility, with negligible cytotoxicity. On the basis of the live‒dead staining results, the EC NPs (<40 μg/mL) exhibited no significant cytotoxicity toward chondrocytes (Fig. 2F, S4). Additionally, flow cytometry analysis confirmed that chondrocytes began to undergo apoptosis at concentrations ≥40 μg/mL (Fig. 2G). Therefore, we selected a concentration of 20 μg/mL for subsequent experiments. Finally, the capacity of chondrocytes to take up EC NPs was observed via confocal microscopy. As shown in Fig. 2H and Fig. S5, within 6 h, a large amount of Cy5.5-labeled EC NPs were taken up by chondrocytes, while only a small amount of Cy5.5-labeled Cys and EGCG were internalized by chondrocytes. This indicates that the self-assembled nanoparticles promote superior chondrocyte uptake.

### 2.3 EC NPs inhibit H_2_O_2_-induced ferroptosis of chondrocytes

**Fig. 3:**
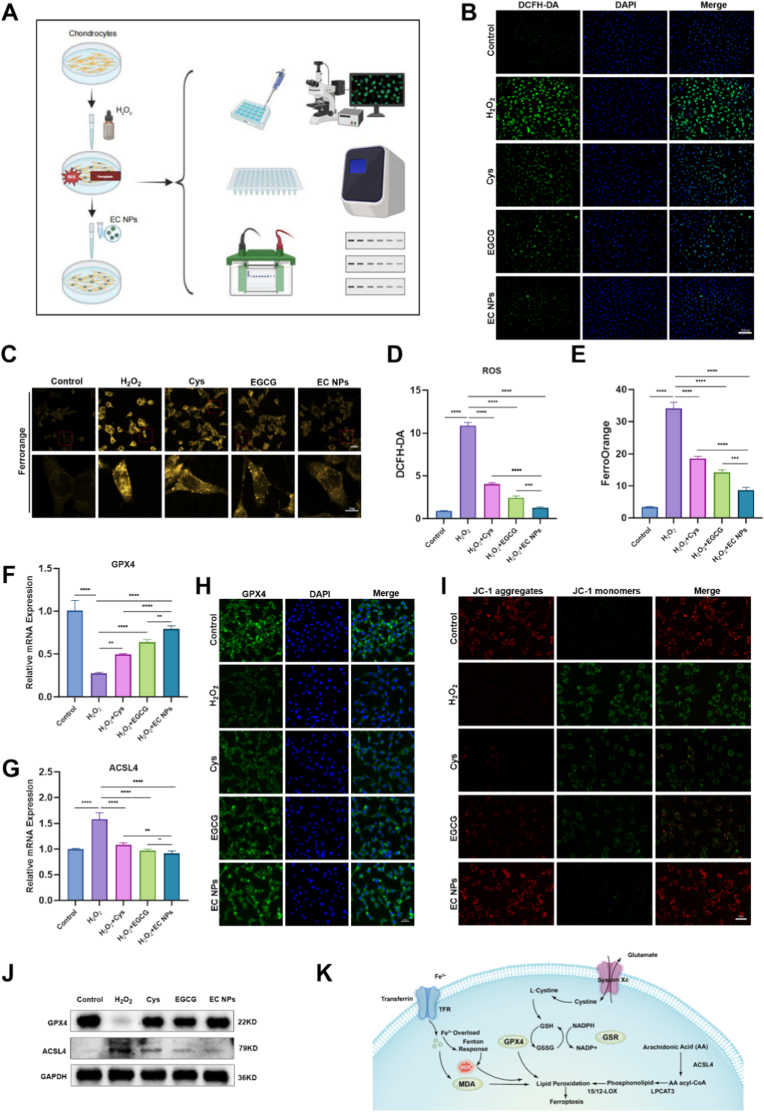
**EC NPs inhibit H2O2-induced ferroptosis of chondrocytes.** (A) H_2_O_2_ stimulation was used to construct a model of oxidative stress and ferroptosis in chondrocytes. (B, D) The expression level of ROS in chondrocytes of each group was assessed using DCFH-DA probe. (C, E) The expression level of Fe2^+^ in chondrocytes of each group was assessed using FerroOrange probe. (F, G) The mRNA expression levels of GPX4 and ACSL4 were quantified using qRT-PCR analysis. (H) Immunofluorescence staining of GPX4 in chondrocytes of each group. (I) JC-1 staining was used to assess changes in mitochondrial membrane potential in chondrocytes of each group. (J) Western blot detection of GPX4 and ACSL4 protein expression level. (K) Schematic diagram of ferroptosis occurring within cells. The error bars indicate the mean ± standard deviation. ns (no statistical significance), ∗P < 0.05, ∗∗P < 0.01, ∗∗∗P < 0.001, ∗∗∗∗P < 0.0001.

To elucidate the role of EC NPs in the inhibition of ferroptosis, we constructed a chondrocyte ferroptosis model by stimulating chondrocytes with H_2_O_2_ (Fig. 3A). Previous studies have suggested that intracellular reactive oxygen species (ROS) can mediate ferroptosis [[28], [29], [30]]. ROS are involved in the regulation of ferroptosis through multiple pathways, in which the accumulation of ROS leads to lipid peroxidation, which in turn disrupts the cell membrane structure and ultimately triggers cell death [29,31]. The major sources of ROS include mitochondrial metabolism, NADPH oxidase (NOX) at the cell membrane, and the Fenton reaction [[32], [33], [34]]. These ROS-generating pathways play important roles in ferroptosis. ROS can promote the peroxidation of polyunsaturated fatty acids to produce lipid peroxides. The accumulation of lipid peroxides is one of the hallmarks of ferroptosis, which leads to cell membrane damage and thus triggers ferroptosis. Moreover, the accumulation of iron generates more ROS through the Fenton reaction, which further exacerbates lipid peroxidation, forming a vicious cycle that promotes the occurrence of ferroptosis [35]. In addition, ROS can affect the process of ferroptosis by regulating intracellular iron metabolism and the antioxidant defense system [36,37]. First, as shown in Fig. 3B and D, to investigate the role of EC NPs in scavenging ROS, we measured ROS levels in the chondrocytes of each group via a DCFH-DA probe and found that ROS (green fluorescence) were significantly elevated in the H_2_O_2_-treated group compared with those in the control group. To distinguish total ROS from lipid ROS, we employed flow cytometry to measure lipid ROS levels detected by the C11-BODIPY probe in chondrocytes across all groups. Results revealed significantly elevated lipid ROS in the H_2_O_2_-treated group. Following treatment with EGCG, Cys, and EC nanoparticles, intracellular lipid ROS levels decreased significantly. Compared to EGCG and Cys, EC NPs exhibited a more pronounced inhibitory effect on lipid ROS levels (Fig. S6 A and B). The intracellular levels of ROS were significantly reduced by treatment with EGCG, Cys, and EC NPs, and we found that, compared with EGCG and Cys, EC NPs significantly reduced the ROS levels. As a key marker of oxidative stress, the expression level of ferric ions is important for revealing the cellular redox status and ferroptosis process [38]. To investigate the correlation between iron ion levels in chondrocytes and ROS-mediated ferroptosis, we examined iron ion levels in each group of chondrocytes via FerroOrange staining. As shown in Fig. 3C and E, our results revealed that the accumulation of Fe^2+^ (orange fluorescence) significantly increased after H_2_O_2_ treatment compared with that in the control group. The abnormal accumulation of Fe^2+^ in chondrocytes gradually decreased after intervention with EGCG, Cys, and EC NPs, and the effect of EC NPs was greater than that of EGCG and Cys. High-resolution Mitotracker staining also demonstrates that EC NPs aid in restoring mitochondrial dysfunction within chondrocytes (Fig. S7). A malondialdehyde (MDA) assay kit was subsequently used to detect the MDA level in each group. As shown in Fig. S8, the expression level of MDA in chondrocytes significantly increased after H_2_O_2_ treatment, and the level of MDA in chondrocytes significantly decreased after EC NPs intervention. These results further confirmed that the nanoparticles could attenuate the H_2_O_2_-induced accumulation of ROS and Fe^2+^ in chondrocytes and play an important role in inhibiting ferroptosis in chondrocytes. Next, we detected ferroptosis-related marker genes at the genetic level via qPCR. As shown in Fig. 3F–G, compared with those in the control group, H_2_O_2_ treatment significantly reduced the mRNA expression level of ferroptosis protection factor (GPX4) and increased the mRNA expression levels of ACSL4. The mRNA expression level of GPX4 was significantly increased, and the mRNA expression level of ACSL4 was significantly decreased after treatment with EGCG, Cys and EC NPs, in which EC NPs were more effective. Immunofluorescence images and quantitative results also revealed that EC NPs reversed the reduction in GPX4 induced by H_2_O_2_ intervention (Fig. 3H, Fig. S9). As shown in Fig. 3J and Figs. S11 and 12, Western blot (WB) and quantitative analysis results similarly demonstrated that H_2_O_2_ intervention led to decreased expression levels of GPX4 and FTH1 proteins while increasing ACSL4 and TFRC protein expression levels. Conversely, treatment with EGCG, Cys, and EC nanoparticles significantly restored GPX4 and FTH1 expression levels and markedly downregulated ACSL4 and TFRC protein expression levels. Furthermore, the EC nanoparticle group exhibited the highest GPX4 and FTH1 expression levels and the lowest ACSL4 and TFRC expression levels. These results further demonstrate the significant role of the nanoparticles in inhibiting ferroptosis in chondrocytes. Finally, to assess the mitochondrial functional status, we measured the mitochondrial membrane potential in each group via a JC-1 detection kit. As shown in Fig. 3I and Fig. S10, H_2_O_2_ treatment significantly decreased the mitochondrial membrane potential of chondrocytes compared with that of the control group. In contrast, EGCG, Cys and EC NP interventions significantly ameliorated the loss of the mitochondrial membrane potential in chondrocytes. Among them, the mitochondrial membrane potential was most significantly restored after EC NPs intervention. According to the above experiments, we hypothesized that EC NPs blocked the vicious cycle of lipid peroxidation and the Fenton reaction by scavenging ROS and decreasing iron ion levels. Moreover, they upregulate GPX4 expression and downregulate ACSL4 to increase antioxidant capacity and inhibit ferroptosis, restore the mitochondrial membrane potential, maintain mitochondrial functional homeostasis, and reduce ROS generation, forming a positive feedback mechanism, thus synergistically inhibiting the progression of ferroptosis in multiple dimensions (Fig. 3K).

### 2.4 EC NPs ameliorate the H_2_O_2_-induced inflammatory response in chondrocytes

**Fig. 4:**
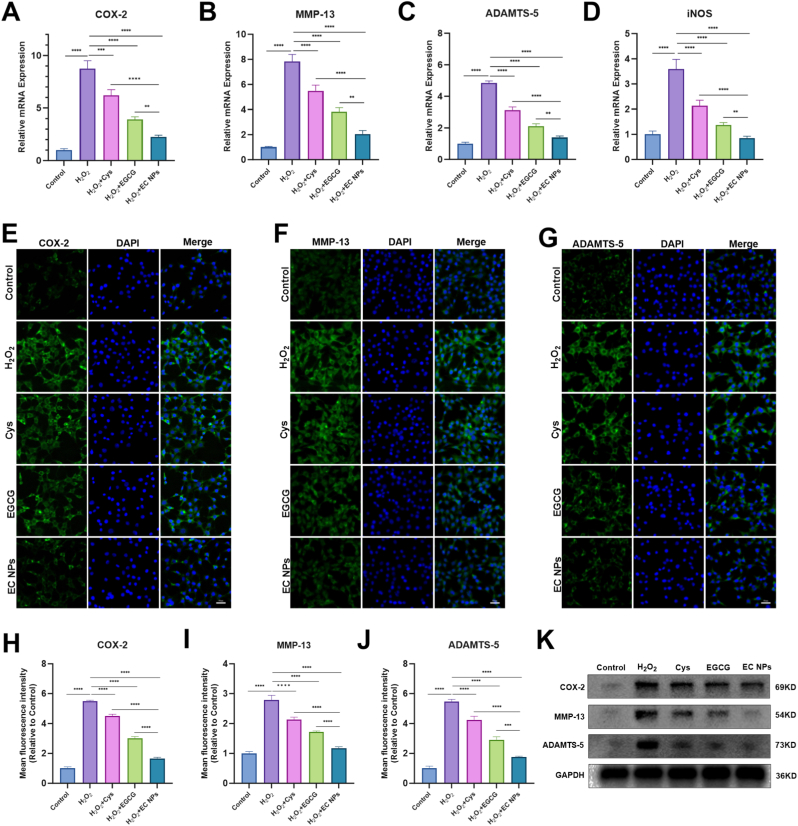
**EC NPs ameliorate H2O2-induced inflammatory response in chondrocytes.** (A–D) Quantification of mRNA expression levels of COX-2, MMP-13, ADAMTS-5, and iNOS by qRT-PCR analysis. (E–G) Immunofluorescence staining was used to assess the expression levels of COX-2, MMP-13, and ADAMTS-5 in chondrocytes of each group. (H–J) Quantitative analysis of COX-2, MMP-13, ADAMTS-5 fluorescence intensity in chondrocytes of each group. (K) Western blot detection of COX-2, MMP-13, ADAMTS-5 protein expression levels. The error bars indicate the mean ± standard deviation. ns (no statistical significance), ∗P < 0.05, ∗∗P < 0.01, ∗∗∗P < 0.001, ∗∗∗∗P < 0.0001.

Previous studies have shown that the abnormal accumulation of iron ions in chondrocytes induces ferroptosis while activating inflammatory responses [39,40]. The accumulation of iron ions generates reactive oxygen species (ROS) via the Fenton reaction, which promotes lipid peroxidation and damage to cell membranes, triggering ferroptosis [41,42]. This process releases damage-associated molecular patterns (DAMPs), which activate inflammatory vesicles and stimulate the release of inflammatory mediators such as cyclooxygenase-2 (COX2) and tumor necrosis factor-α (TNF-α), exacerbating the inflammatory response [43]. We continued to use H_2_O_2_ to establish a chondrocyte inflammation induction model to further investigate the effects of EC NPs on chondrocyte inflammatory responses. The expression levels of inflammatory factors in each group were analyzed by qPCR and immunofluorescence after treatment with EGCG, Cys, or EC NPs. As shown in the qPCR results in Fig. 4A–D, the mRNA expression of the inflammatory factors COX-2, MMP-13, ADAMTS-5, and iNOS increased after H_2_O_2_ treatment. In contrast, the mRNA expression of inflammatory factors was significantly decreased after the interventions with EGCG, Cys and EC NPs. Although all three interventions reduced the expression of inflammatory factors, compared with EGCG and Cys, EC NPs significantly reduced the mRNA expression levels of the inflammatory factors COX-2, MMP-13, ADAMTS-5, and iNOS. As shown in Fig. 4E–G, the immunofluorescence results revealed that the fluorescence of COX-2, MMP-13 and ADAMTS-5 was significantly enhanced in H_2_O_2_-treated chondrocytes, and the fluorescence intensity was significantly weakened after intervention with EGCG, Cys and EC NPs, in which the fluorescence intensity was significantly lower in the intervention group of EC NPs than in the EGCG and Cys groups. As shown in Fig. 4H–J, the results of the quantitative fluorescence analysis confirmed the above results. Finally, we used WB analysis to assess the protein expression levels of COX-2, MMP-13 and ADAMTS-5. As shown in Fig. 4K and Fig. S13, treatment with EGCG, Cys, and EC NPs significantly reduced the H_2_O_2_-induced protein levels of COX-2, MMP-13, and ADAMTS-5, with EC NPs showing the most pronounced inhibitory effect on the expression of these inflammatory factors. These findings indicate that EC NPs have a marked effect on alleviating the inflammatory response of chondrocytes.

### 2.5 EC NPs ameliorate H_2_O_2_-induced metabolic disturbances in chondrocytes

**Fig. 5:**
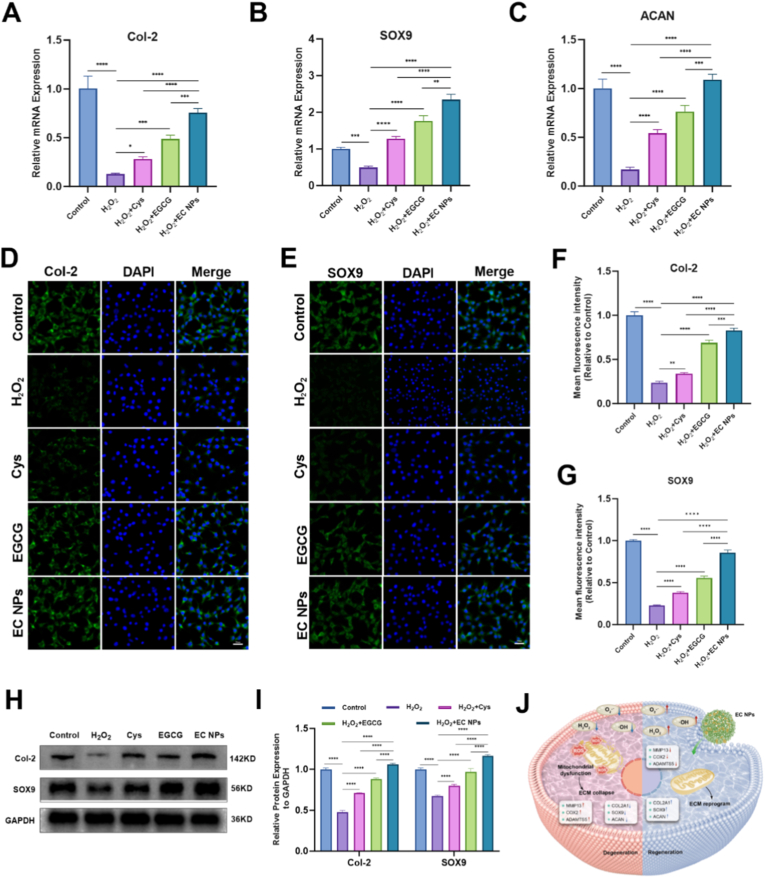
**EC NPs ameliorate H2O2-induced metabolic disturbances in chondrocytes.** (A–C) Quantification of mRNA expression levels of Col-2, SOX9 and ACAN by qRT-PCR analysis. (D, F) Immunofluorescence staining and quantification of the fluorescence intensity of Col-2 in chondrocytes of each group. (E, G) Immunofluorescence staining and quantitative analysis of the fluorescence intensity of SOX9 in chondrocytes of each group. (H) Western blot for Col-2 and SOX9 protein expression. (I) Relative expression of Col-2 and SOX9 proteins. (J) Schematic illustration: Chondrocyte uptake of EC NPs alleviates oxidative stress and restores mitochondrial homeostasis to reprogram the extracellular matrix. The error bars indicate the mean ± standard deviation. ns (no statistical significance), ∗P < 0.05, ∗∗P < 0.01, ∗∗∗P < 0.001, ∗∗∗∗P < 0.0001.

Previous studies have shown that abnormal aggregation of iron ions can catalyze the production of ROS, leading to elevated intracellular ROS levels, which may interfere with normal cellular metabolic processes and result in decreased expression levels of factors related to the synthesis and maintenance of the extracellular matrix (such as SOX9, COL2A1, and ACAN) [[44], [45], [46]]. Downregulation of these factors impairs the ability of chondrocytes to synthesize and repair the extracellular matrix, accelerates degradation of the extracellular matrix, and promotes the development of osteoarthritis (OA). As shown in Fig. 5A–C, H_2_O_2_ inhibited the mRNA expression of SOX9, COL2A1 and ACAN. After treatment with EGCG, Cys, and EC NPs, the H_2_O_2_-induced downregulation of SOX9, COL2A1, and ACAN mRNA expression was significantly reversed, with EC NPs showing the most pronounced restorative effect. As shown in Fig. 5D–E, the immunofluorescence and quantitative analysis of SOX9 and COL2A1 revealed that the fluorescence intensity of SOX9 and COL2A1 was significantly reduced after H_2_O_2_ treatment, whereas treatment with EGCG, Cys, and EC NPs markedly restored their expression, with EC NPs showing a more pronounced effect than EGCG or Cys alone. To further validate the role of EC NPs in improving this aspect of chondrocyte metabolic disorders, the protein expression levels of Col-2 and SOX9 were analyzed by Western blotting. As shown in Fig. 5H–I, the protein expression levels of Col-2 and SOX9 were significantly decreased after H_2_O_2_ induction, while EC NPs had a significant advantage in alleviating the H_2_O_2_-induced decrease in the protein expression levels of SOX9 and Col-2, which indicated that EC NPs effectively alleviated the metabolic disorders of the cartilage matrix. These findings suggest that EC NPs can effectively maintain the homeostasis of the cartilage matrix, inhibit the degradation of the extracellular matrix, and inhibit the development of osteoarthritis (Fig. 5J).

### 2.6 Transcriptomics of EC NPs in H_2_O_2_-treated chondrocytes

**Fig. 6:**
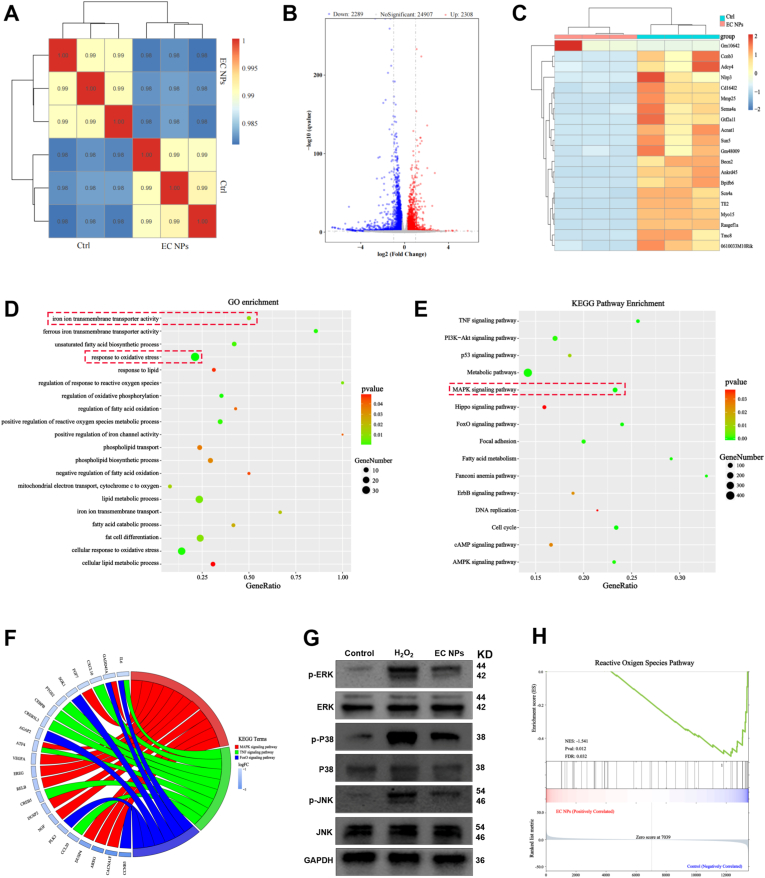
**Transcriptomic changes after EC NPs intervention in H2O2-Treated chondrocytes.** (A) Heatmap of sample-to-sample distance. (B) Volcano plot showing differential gene expression in the H_2_O_2_-stimulated control and EC NPs groups. (C) Heatmap of different genes (TOP50) in the control and EC NPs groups stimulated by H_2_O_2_. (D) GO enrichment analysis of DEGs between the two groups. (E) KEGG pathway enrichment analysis of DEGs between the two groups. (F) KEGG enrichment chordal graph. (G) Western blotting analysis of p-ERK, p-P38, and p-JNK expression levels relative to ERK, P38, and JNK expression levels in chondrocytes treated with H_2_O_2_ stimulation in the control group and EC NPs group. (H) Reactive oxygen species pathway based on GSEA analysis. The error bars indicate the mean ± standard deviation. ns (no statistical significance), ∗P < 0.05, ∗∗P < 0.01, ∗∗∗P < 0.001, ∗∗∗∗P < 0.0001.

To elucidate the mechanism of action of EC NPs on chondrocytes and the differences in gene expression between EC NPs and controls, we performed RNA sequencing (RNA-seq), and replicates of three independent experiments produced consistent results (Fig. 6A). Differentially expressed genes (DEGs) were screened based on the thresholds |log2 FC| > 1 and p-value <0.05. The volcano plot of differentially expressed genes revealed that, compared to the control group, 2308 genes were upregulated and 2289 genes were downregulated in the EC nanoparticle group. Among these, GPX4, Col2a1, and SOX9 genes showed upregulation, while inflammation-related genes (MMP13, MMP3, and Adamts5) exhibited downregulation (Fig. 6B). All differentially expressed genes are shown in the heat map, with the top 50 differentially expressed genes highlighted (Fig. S14 and Fig. 6C). As shown in Fig. 6D, GO enrichment analysis revealed that the DEGs identified after EC NPs intervention were associated mainly with oxidative stress, iron ion homeostasis and lipid metabolic processes. Kyoto Encyclopedia of Genes and Genomes (KEGG) enrichment analysis of differentially expressed genes was also performed, which revealed that signaling pathways such as the MAPK, TNF, and p53 pathways were downregulated in chondrocytes treated with EC NPs. Chord plot analysis further revealed that EC NPs affected both the MAPK signaling pathway and the TNF signaling pathway (Fig. 6F). Subsequently, we further validated the MAPK signaling pathway through Western blotting and found that H_2_O_2_ significantly upregulated the expression of phosphorylated ERK, p38, and JNK in chondrocytes. However, EC NPs treatment significantly reduced the expression of phosphorylated ERK, p38, and JNK (Fig. 6G). Therefore, the data indicate that EC NPs protect H_2_O_2_-stimulated chondrocytes from further inflammatory progression by inhibiting the MAPK signaling pathway. Gene set enrichment analysis (GSEA) results also confirmed that differentially expressed genes (DEGs) were enriched in pathways such as reactive oxygen species (ROS) and p53 (Fig. 6H, Fig. S15). Our results suggest that, compared with H_2_O_2_ treatment, EC NPs treatment may have suppressed the expression of genes related to iron ion homeostasis, oxidative stress, the inflammatory response, and lipid metabolism; inhibited inflammation and progression by downregulating signaling pathways such as the MAPK, TNF, and p53 pathways; and ultimately attenuated cartilage degeneration and slowed the progression of osteoarthritis.

**Fig. 7:**
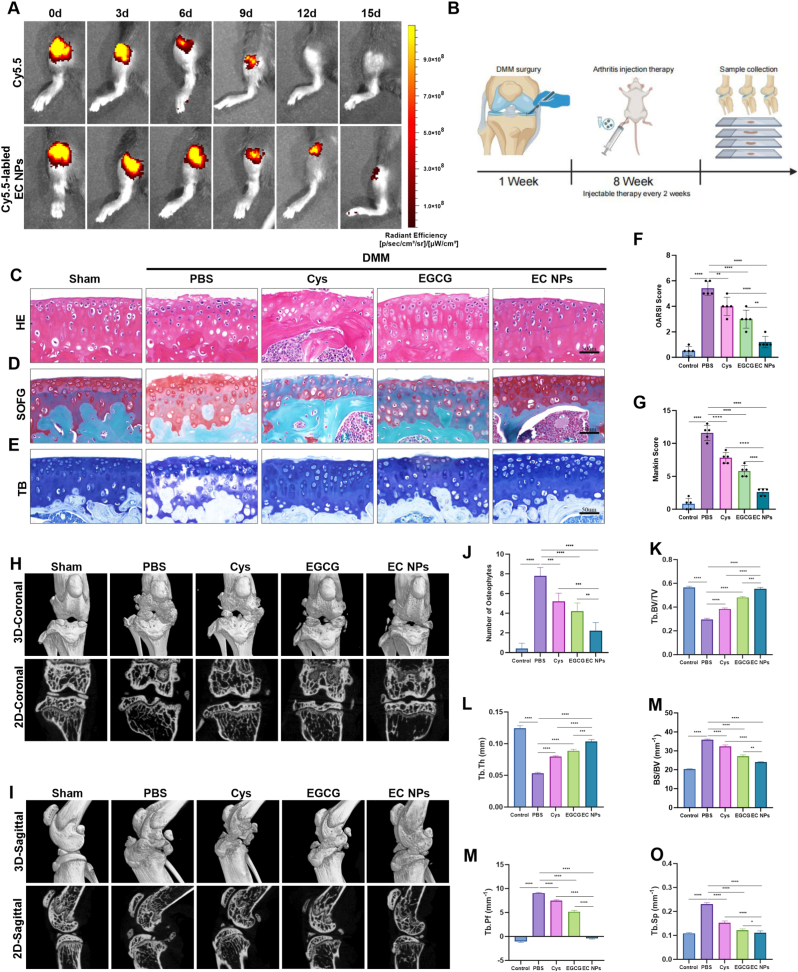
**EC NPs alleviate DMM-induced OA in mice.** (A) Representative fluorescence images of Cy5.5 dye and Cy5.5-labeled EC NPs injected into the knee joint. (B) Schematic diagram of the experimental design of the DMM-induced OA mouse model, intra-articular injection and sample collection. (C–E) HE staining, SO-FG staining and TB staining of knee joint sections in each group. (F) OARSI score. (G) Mankin score. (H, I) Representative coronal and sagittal CT images of the knee joints of each group. (J) The number of osteophytes in each group. (K–O) Measurement of subchondral bone microstructure in the tibia to assess the degree of knee joint degeneration. The error bars indicate the mean ± standard deviation. ns (no statistical significance), ∗P < 0.05, ∗∗P < 0.01, ∗∗∗P < 0.001, ∗∗∗∗P < 0.0001.

### 2.7 EC NPs alleviate DMM-induced OA in mice

To determine the interval of drug administration, we first evaluated the drug retention time of the EC NPs in the knee joint cavity of the mice. As shown in Fig. 7A, two groups of mice were selected, Cy5.5 fluorescent dye and Cy5.5-labeled EC NPs were injected into their joint cavities, and small-animal in vivo imaging was performed to monitor the intensity of the fluorescence signals at days 0, 3, 6, 9, 12, and 15. By quantitative analysis of the fluorescence intensity in the infrared imaging map, we found that the fluorescence in the Cy5.5 fluorescent dye alone group completely disappeared on day 12, whereas the knee joints of the mice in the Cy5.5-labeled EC NPs group still retained fluorescent signals on day 12, and the fluorescence almost disappeared by approximately day 15, which indicated that the EC NPs had a better retention ability and helped us to determine the dosing interval time (Fig. S16). After this, we used the DMM model to establish a mouse model of knee osteoarthritis, and after 1 week of successful modeling, we injected EGCG, Cys, and EC NPs into the joint cavity every 2 weeks for treatment. We recorded changes in body weight across all mouse groups and found no significant differences in weight among them (Fig. S17). Then, we sacrificed the mice after 8 weeks and collected knee specimens from each group. The cartilage tissues of each group were stained with hematoxylin-eosin (HE), saffron O-fixed green (SOFG), and toluidine blue (TB) to evaluate the in vivo biological effects of EC NPs on OA in mice (Fig. 7B). Furthermore, major organs (heart, liver, spleen, lungs, and kidneys) were harvested eight weeks post-surgery for hematoxylin and eosin (HE) staining. No inflammatory infiltration, necrosis, fibrosis, or histological structural abnormalities were detected in any organ, clearly confirming that intra-articular injection of EC-NPs does not induce systemic toxicity (Fig. S18). As shown by the histologic images in Fig. 7C, very severe osteoarthritis occurred in the PBS group compared with the sham-operated group; in the PBS group, the integrity of the cartilage of the mouse knee joints was disrupted, and the surface became rough and even fissured and abraded. The arrangement of the chondrocytes was no longer neat, some of the cells exhibited a swollen or necrotic morphology, and the staining intensity of the extracellular matrix also changed, indicating the loss or degradation of matrix components. Compared with those in the PBS group, the EGCG, Cys and EC NPs treatments all had therapeutic effects but not consistently. Compared with those of the PBS group, the degree of roughness of the surface of the cartilage of the mouse knee joints, the degree of wear and the degree of disorganization of the cellular arrangement in the EGCG and Cys groups improved, whereas the surface of the articular cartilage of the EC NPs group was smooth, with the cells aligned neatly and with normal staining of the matrix. As shown in Fig. 7D–E, the SO-FG and TB staining results revealed severe knee cartilage damage, loss of cartilage surface integrity, reduced cartilage glycosaminoglycan content, matrix destruction, and disorganized distribution of chondrocytes in the PBS group. Compared with those in the PBS group, the pathological changes in the knee joints in the EGCG, Cys, and EC NPs intervention groups improved, and the integrity of the articular cartilage surface integrity was locally lost after EGCG, Cys treatment, chondroitin glycosaminoglycan content, and chondrocyte distribution. Although there was some improvement, the articular surface still exhibited localized cracking, partial destruction of the matrix, and reduced chondroitin glycosaminoglycan content. We did not observe obvious cracks on the surface in the EC NP group, indicating that the articular cartilage was intact in the EC NPs group, and the chondroitin glycosaminoglycan content and cell distribution in this group were similar to those in the sham operation group. As shown in Fig. 7F–G, we calculated OARSI scores and Mankin scores for each group on the basis of the histopathological staining results and found that the scores of the PBS group were significantly greater than those of the sham-operated group, and the scores of each group were significantly lower after the intervention with EGCG, Cys and EC NPs, with a significant reduction in the scores of the EC NPs group compared with those of the EGCG and Cys groups. These findings suggest that EC NPs have significant therapeutic effects on knee osteoarthritis in mice. We subsequently used micro-CT to reconstruct the knee joints of the mice in each group to assess the progression of arthritis in each group, as shown in the representative 2D and 3D reconstructed images of the knee joints in Fig. 7H–I. The articular cartilage surface of the sham-operated group was smooth, and the arrangement of the bone trabeculae was tight and uniformly distributed, indicating good bone density and bone microarchitecture. The joint gap was kept constant and clear, showing the integrity of the cartilage coverage, and no abnormal manifestations, such as osteoid formation or bone erosion, were observed. In contrast, in the PBS group, we observed disorganized trabecular arrangement and increased bone density in some areas, indicating bone sclerosis. The joint space was narrowed, and the subchondral bone showed erosion and cystic degeneration. In some areas, obvious trabecular disorganization was observed, leading to a rough and irregular joint contour. These results confirmed the successful establishment of the mouse OA model. Although the knee joints of the mice treated with EGCG, Cys and EC NPs presented a decrease in bone complexity, an increase in the joint space, a recovery of the trabecular arrangement, and an improvement in the osteosclerosis of the subchondral bone of the knee joint, compared with those of the EGCG and Cys groups, the proliferation of bony structures in the knee joints of the mice in the EC NPS group was markedly reduced, with the trabecular arrangement becoming normalized, a more uniform distribution of bone density, and the osteosclerosis phenomenon in which the bone density distribution was more uniform, and osteosclerosis was alleviated. The joint space was widened, erosion and cystic degeneration of the subchondral bone were reduced, subchondral bone erosion was effectively alleviated, and the joint contour became smoother. Overall, the bone microstructure of the knee joint is well repaired, and the pathological characteristics are significantly improved and close to those of the normal state. We also evaluated the number of osteophyte as well as microstructural parameters of the subchondral bone of the tibia in each group to assess osteoarthritis-related remodeling. The parameters analyzed for microstructural analysis included the trabecular thickness (Tb.Th), bone surface area-to-volume ratio (BS/BV), trabecular type factor (Tb.Pf), bone volume-to-tissue volume ratio (BV/TV), and trabecular spacing (Tb.Sp). As shown in Fig. 7J-O, the results revealed that the mice in the DMM surgery and sham surgery groups exhibited significant microstructural changes, and the analysis of the microstructural results revealed that EC NPs intervention improved subchondral bone parameters. In conclusion, all of the above results confirmed the significant improvement in osteoarthritic knee joints in mice treated with EC NPs. These results not only reflect the effectiveness of EC NPs in alleviating the pathological features of osteoarthritis but also indicate their potential to promote bone microstructure reconstruction and maintain joint stability.

### 2.8 EC NPs alleviate DMM-induced ferroptosis and cartilage inflammation in mouse OA

**Fig. 8:**
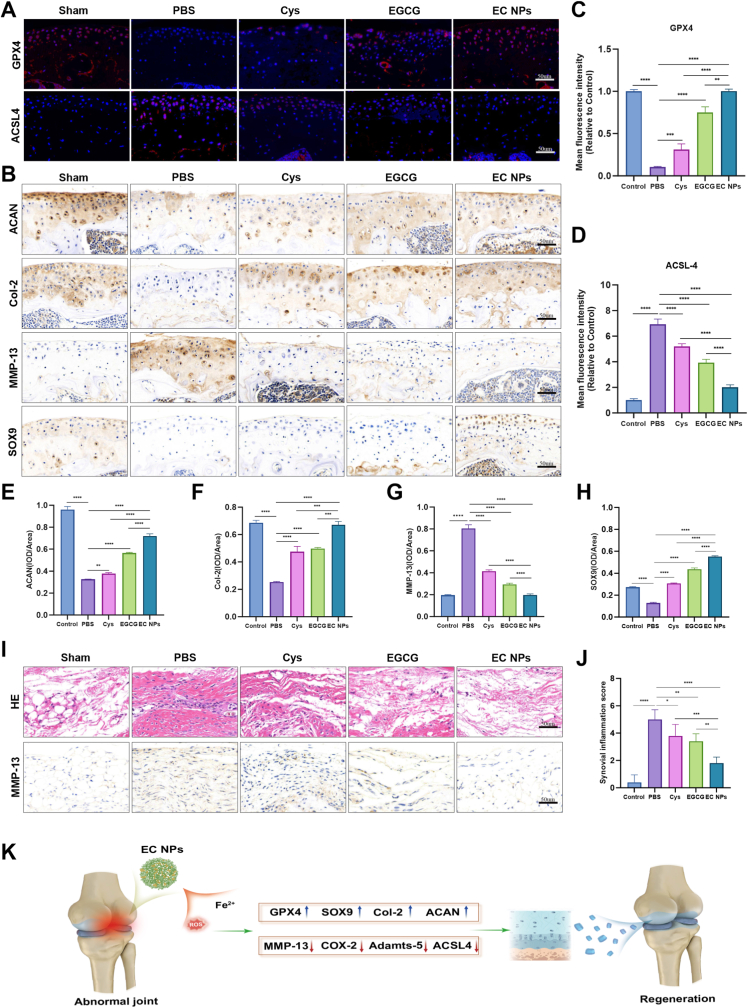
**EC NPs alleviate DMM-induced ferroptosis and cartilage inflammation in mouse OA.** (A) Immunofluorescence staining images of GPX4 and ACSL4 in knee joint sections of each group. (B) Immunohistochemical staining images of ACAN, Col-2, MMP-13 and SOX9 in knee joint sections of each group. (C) Quantification of GPX4 fluorescence intensity in each group. (D) Quantification of ACSL4 fluorescence intensity in each group. (E–H) Results of statistical analysis of protein expression by immunohistochemical staining of ACAN, Col-2, MMP-13 and SOX9. (I) HE-stained synovial tissue from each group of mice. (J) Assessment of synovial inflammation (n = 5). (K) Schematic illustration of the mechanism by which EC NPs alleviate cartilage damage in OA mice. The error bars indicate the mean ± standard deviation. ns (no statistical significance), ∗P < 0.05, ∗∗P < 0.01, ∗∗∗P < 0.001, ∗∗∗∗P < 0.0001.

We further investigated the mechanism of action of the EC NPs in the treatment of OA. The ferroptosis signaling pathway plays a crucial role in regulating the onset and progression of OA. As shown in Fig. 8A, immunofluorescence staining revealed that the expression of GPX4 was significantly downregulated and that of ACSL4 was significantly upregulated in the PBS group compared with the sham operation group. Compared with EGCG and Cys, EC NPs intervention significantly upregulated GPX4 and downregulated ACSL4. The results of the quantitative analysis of the immunofluorescence data shown in Fig. 8C–D also supported these findings. These results further confirmed that EC NPs regulate the ferroptosis signaling pathway in vivo to protect chondrocytes and effectively slow the progression of osteoarthritis. To subsequently verify the biofunctional ability of the EC NPs in vivo, we performed immunohistochemical staining of the tissue samples to assess the protein expression levels of ACAN, Col2a1, MMP-13 and SOX9 in the cartilage region in the joints of each group. As shown in Fig. 8B, immunohistochemistry revealed that the expression of ACAN and Col2a1 was significantly lower in the PBS group than in the sham operation group and that the expression of ACAN, Col2a1 and SOX9 was significantly restored after EC NPs intervention. In the MMP-13 immunohistochemical images, the PBS group presented with dark brown staining, whereas the sham-operated and EC NPs groups presented with light brown staining. As shown in Fig. 8E–H, analysis of the immunohistochemical staining results indicated that EC NPs slowed the progression of osteoarthritis by inhibiting cartilage degradation and clearing inflammatory factors. As shown in Fig. 8I–J, the synovial membranes of the knees of mice in each group were stained with HE and inflammation scores were assessed. The results indicated that the number of synovial membrane endothelial cells in the knees of mice treated with PBS, EGCG, or Cys significantly increased, with synovial tissue proliferation, inflammatory cell infiltration, and significantly higher inflammation scores compared to the sham surgery group. In contrast, the EC NPs group showed less synovial tissue proliferation and no significant increase in inflammation scores. This suggests that EC NPs have an anti-inflammatory effect on injured knee joints. Overall, these findings suggest that EC NPs effectively inhibit ferroptosis, eliminate inflammation, and protect cartilage from degradation, thereby mitigating the progression of OA.

## 3 Discussion

This study has several limitations. First, the experiment exclusively utilized the ATDC5 immortalized cell line, whose phenotype differs from that of adult primary chondrocytes. Subsequent work should incorporate human-derived chondrocytes and fresh cartilage tissue blocks to validate the uptake, metabolism, and GAG retention capacity of EC-NPs in human systems. Second, the study employed only a mouse DMM model with a limited sample size. Future research should expand the cohort and incorporate large animal models such as sheep or pigs to validate the reproducibility of efficacy and safety in larger joints. Additionally, validation of the ferroptosis pathway currently relies solely on GPX4, ACSL4, and ROS levels, lacking systematic evidence from mitochondrial morphology, iron metabolism tracking, and multi-omics approaches. Concurrently, attention must be paid to the long-term biocompatibility of EC nanoparticles, the metabolic pathways of degradation products, and potential immunogenicity. These limitations indicate the need for validation in large animal models, human tissue microarrays, and multi-omics platforms, alongside pharmacokinetic/toxicological studies to advance clinical translation. These limitations should be addressed in future studies to facilitate clinical translation.

## 4 Conclusion

In summary, we report a nanoparticle for the treatment of OA that inhibits ferroptosis to alleviate the inflammatory response and correct the imbalance in chondrocyte matrix metabolism. EC NPs exhibit good biocompatibility and stability, which ensures that they can be sustained in vivo. EC NPs inhibit chondrocyte ferroptosis through the elimination of ROS and a reduction in iron loading, attenuate the inflammatory response of chondrocytes, correct the metabolic imbalance in the chondrocyte cell matrix, and promote cartilage anabolism, thus maintaining metabolic homeostasis. In addition, it inhibits the continuous release of inflammatory factors by chondrocytes during inflammatory responses by suppressing the MAPK and TNF signaling pathways. In vivo experiments also confirmed that EC NPs effectively alleviated periarticular bone remodeling and mitigated OA progression. In conclusion, our study strongly supports the potential of EC NPs in alleviating OA by inhibiting ferroptosis, indicating substantial advancements in OA therapeutic strategies.

## CRediT authorship contribution statement

**Zhao Lin:** Conceptualization, Data curation, Formal analysis, Funding acquisition, Investigation, Methodology, Project administration, Resources, Software, Supervision, Validation, Visualization, Writing – original draft, Writing – review & editing. **Jiayao Zhang:** Conceptualization, Data curation, Formal analysis, Investigation, Methodology, Validation, Visualization, Writing – original draft. **Peng Zhang:** Conceptualization, Data curation, Project administration, Visualization, Writing – original draft. **Mingjuan Zhang:** Data curation, Methodology, Resources, Software, Supervision, Validation. **Hanhao Dai:** Data curation, Funding acquisition, Project administration, Supervision. **Yibin Su:** Conceptualization, Data curation, Methodology, Software, Supervision. **Haiqi Ding:** Data curation, Resources, Software, Visualization. **Linhai Yang:** Conceptualization, Formal analysis, Validation. **Guoming Liu:** Data curation, Investigation, Resources, Writing – review & editing. **Jie Xu:** Data curation, Funding acquisition, Software, Validation, Visualization, Writing – review & editing. **Jun Luo:** Conceptualization, Data curation, Formal analysis, Funding acquisition, Investigation, Methodology, Project administration, Resources, Software, Supervision, Validation, Visualization, Writing – original draft, Writing – review & editing.

## Declaration of competing interest

The authors declare no conflict of interest.

## Data Availability

No data was used for the research described in the article.
