# Supplementary material for: Supramolecular self-assembly of EGCG–cysteine nanodrugs for ferroptosis and oxidative stress inhibition in chondrocytes to treat osteoarthritis
Source: Mater Today Bio. 2026 Mar 10;38:102978. doi: 10.1016/j.mtbio.2026.102978 (PMC13019579; doi:10.1016/j.mtbio.2026.102978)
Supplement: Multimedia component 1 [file mmc1.docx]

**Supporting Information**

**Supramolecular Self-Assembly of EGCG–Cysteine Nanodrugs for Ferroptosis and Oxidative Stress Inhibition in Chondrocytes to Treat Osteoarthritis**

1. **Materials and methods**

*1.1. Materials:*

Epigallocatechin-3-gallate (EGCG), cysteine (Cys) and formaldehyde were purchased from Aladdin Biochemicals Technology Co. 1,1-Diphenyl-2-picrylhydrazyl (DPPH, 1707-751) and hydrogen peroxide (H_2_O_2_, 88,597-100 ML-F, 3 wt %) were purchased from Sigma-Aldrich (China). 2′,7′-Dichlorodihydrofluorescein diacetate (DCFH-DA) was purchased from Sevier Bio (Wuhan, China). Cellular Ferrous Ion Detection Fluorescent Probe (FerroOrange, F374) was purchased from Dojindo (Japan). Hoechst 33342 (C1022), Cell Counting Kit 8 (CCK8) (C0037), Lipid Oxidation (MDA) Detection Kit (S0131S), Hematoxylin-Eosin (HE) Staining Kit (C0105S), 2,2′-azinobis(3-ethylbenzothiazoline-6-sulfonic acid) diammonium salt (ABTS, Y083341), 2-phenyl-4,4,5,5-tetramethylimidazoline-1-oxo 3-oxide (PITO, Y091473), Safranin O-Fixed Green (SO-FG) cartilage staining kit (C0621S), Toluidine Blue (TB) staining solution (C0637) were purchased from Beyotime Biotechnology (Shanghai, China).

*1.2. Synthesis of EC NPs:*

EGCG (0.4 mmol) was dissolved in deionized water (35 mL), and then 60 μL of formaldehyde was added at room temperature (25◦ C). 5 mL of Cys aqueous solution (0.4 mmol) was added after 5 min, and the clear solution gradually became opaque after stirring for about 30 min. Finally, the EC NPs were purified by centrifugation (10,000 r min-1 , 10 min), collecting the NPs and washing with deionized water three times.

*1.3. Measurements:*

The morphology of EC NPs was observed using a scanning electron microscope (SEM, Sigma 300; ZEISS, Germany) and a transmission electron microscope (TEM, Tecnai G2 F20; Thermo Fisher, USA). The dimensions of EC NPs were measured by dynamic light scattering (DLS, Zetasizer Nano ZS90, UK). Elemental maps were obtained by energy dispersive X-ray spectroscopy (EDS, Sigma 300; ZEISS, Germany). X-ray photoelectron spectroscopy (XPS) was evaluated by Thermo Fisher ESCALAB 250Xi (USA). Fourier transform infrared spectra (FT-IR) were evaluated by SHIMADZU IRTracer-100 (Japan). Mass spectra of EC NPs were obtained by Bruker autoflex speed (USA) MALDI-TOF/TOF in CHCA matrix reflectance mode. The X-ray diffraction (XRD) patterns of EC NPs, EGCG, and cysteine were determined using an advanced powder diffractometer (Bruker D8, Bruker Corporation, USA). UV-Vis spectra were recorded by a UV spectrophotometer (SHIMADZU-UV-2600 I, Japan).

*1.4. Antioxidant Ability of EC NPs:*

The DPPH radical scavenging assay was performed as in previous studies. First, 700 μL of DPPH radical working solution (0.1 mM) was mixed with 100 μL of EC NPs solution at different concentrations (5, 10, 20, 40 μg/mL). The mixtures were stored in the dark at 25 °C for 30 min. The absorbance was measured at 517 nm using a UV spectrophotometer. Then, the mixed solution was placed in the dark and the absorption was detected at a predetermined time point. DPPH radical scavenging capacity was calculated as S = [1 - (Am - An)/A0] × 100%, where Am is the absorbance of the DPPH resuspension with EC NPs, An is the absorbance of EC NPs in ethanol solution, and A0 is the absorbance of the DPPH solution without EC NPs. The ABTS and PTIO methods were used to detect the total antioxidant capacity and nitrogen oxide radical scavenging capacity, respectively.

*1.5. Cell Viability Assay:*

CCK8 was used to detect the cell viability of EC NPs. ATDC5 was incubated with DMEM medium containing different concentrations of EC NPs (2.5, 5, 10, 20, 40, 80 μg/mL) in 96-well plates for 1 and 2 days. Pure medium was used as a control group. After 1 and 2 days of incubation, all medium in each well was replaced with 10 μL of CCK8 and 90 μL of DMEM medium and stored in the dark at 37 °C for 2 hours. The absorbance values at 450 nm were subsequently determined using an enzyme marker (VARIOSKAN FLASH, Thermo Fisher scientific, USA).

*1.6. Live/Dead Staining:*

ATDC5 was incubated with DMEM medium containing different concentrations of EC NPs in 24-well plates for 1 day and then stained with a live/dead cell double staining kit. After being kept in the dark for 20 min, the cells were rinsed three times with PBS and then observed under an inverted fluorescence microscope (Leica, DMI4000 B; Germany).

*1.7. Cell apoptosis staining:*

Flow cytometry was used to analyze chondrocyte apoptosis. Cells were cultured with different concentrations of EC NPs for 24 h. After incubation, the cells underwent trypsinization. For apoptosis analysis, the Annexin V-FITC apoptosis detection kit (C1062S, Beyotime, China) was used, following the manufacturer's instructions. Flow cytometry analysis of stained cells was performed using FlowJo software (version 10.4) to process data according to standard procedures.

*1.8. Intracellular uptake of EC NPs:*

For the cellular uptake study, ATDC5 cells were incubated with Cy5.5-labeled Cys, Cy5.5-labeled EGCG, or Cy5.5-labeled EC NPs for 6 h, followed by three washes with phosphate-buffered saline (PBS). Chondrocytes were fixed with 4% paraformaldehyde (G1101, Servicebio, China) for 15 min and incubated in 0.5% Triton X-100 (T9284, Sigma, USA) for 15 min. After washing three more times with PBS, the cells were treated with 100 μM TRITC Phalloidin (CA1610, Solarbio, China) and DAPI (C1006, Beyotime, China) for 20 min. Subsequently, chondrocytes were observed using a laser confocal microscope (Leica, TCS SP8 X; Germany).

*1.9. Intracellular oxidative stress detection:*

ATDC5 were cultured in medium containing 200 μM H₂O₂, with the addition of 20 μg/mL Cys, EGCG and EC NPs for 6 hours. The cells were divided into control, H₂O₂, H₂O₂+Cys, H₂O₂+EGCG, and H₂O₂+EC NPs groups, and the levels of reactive oxygen species (ROS) in the chondrocytes of each group were detected using the DCFH-DA probe. After incubation for 30 min in a 5% CO2 cell incubator at 37°C, the cells were washed three times with serum-free medium and observed under a fluorescence microscope (Leica, DMI4000 B; Germany). Chondrocytes were incubated with JC-1 staining solution and at 37°C for 20 min, then washed three times with JC-1 buffer and visualized under a laser confocal microscope (Leica, TCS SP8 X; Germany). MDA levels were detected in each group using a lipid peroxidation (malondialdehyde, MDA) kit.

*1.10. Fe²⁺ detection:*

24 μg of FerroOrange fluorescent probe was dissolved in 35 μL of dimethylsulfoxide (DMSO), and then diluted with saline to obtain a working solution of 1 μmol L-1. The treated cells were incubated with the working solution for 30 min at 37°C under 5% CO2 without washing and observed under a laser confocal microscope (Leica, TCS SP8 X; Germany).

*1.11. Quantitative real-time polymerase chain reaction analysis:*

Total RNA was extracted from each group using an RNA extraction kit (AG21023, Avery Bio, China). RNA concentration and purity were determined, and the RNA was reverse-transcribed into complementary DNA (cDNA). Quantitative real-time polymerase chain reaction (qRT-PCR) was then performed using an RT-PCR system. The primer sequences used in this study are listed in Table S1.

*1.12. Western blot analysis:*

The levels of relevant proteins were assessed by Western blotting. Chondrocytes were collected from each group and total protein was extracted. After centrifugation at 12,000 g for 20 min at 4°C, the supernatant was poured out and mixed with the sample buffer at a ratio of 4:1. The mixture was then heated to 100°C for 10 min. The target proteins were transferred onto polyvinylidene difluoride (PVDF) membranes (IPVH00010, Millipore, USA) and electrophoresed on 10% sodium dodecyl sulfate-polyacrylamide gels, which were subsequently probed with their corresponding primary antibodies (Table S2). After overnight incubation at 4°C, the membranes were washed with TBS Tween (TBST) buffer three times for 10 min each. Subsequently, PVDF membranes were incubated with secondary antibodies (ZB-5301, zsbio.com, China) for 2 h at room temperature and washed with TBST 3 times for 10 min each. Finally, protein content was displayed using an Invitrogen iBright imaging system (Thermo Fisher, USA).

*1.13. Immunofluorescence staining:*

Chondrocytes were fixed with 4% paraformaldehyde for 20 minutes, permeabilized with 0.5% Triton X-100 for 15 minutes, and then blocked with 5% BSA for 2 hours. Subsequently, rabbit primary antibodies against COX-2, SOX9, ACSL4, GPX4, MMP-13, ADAMTS-5, and COL2A1 were diluted at a ratio of 1:100 and incubated at 4°C for 12 hours. Subsequently, goat anti-rabbit secondary antibody (RGAR001, Proteintech, China) was diluted to a concentration of 1:100 and incubated with chondrocytes for 1 h at room temperature. Then the cells were stained with Hoechst 33342 (diluted 1:1000) for 20 min and finally visualized by high-content screening (Opera Phenix Plus, PerkinElmer, USA).

*1.14. Transcriptome sequencing:*

Total RNA was extracted using Trizol reagents (AG, China) according to the manufacturer's protocols, and RNA purity and quantification were assessed using a NanoDrop 2000 Spectrophotometer (Thermo Scientific, USA). RNA integrity was assessed by an Agilent 2100 Bioanalyzer (Agilent Technologies, USA). Libraries were constructed using the VAHTS Universal V6 RNA-seq Library Preparation Kit according to the manufacturer's instructions. Transcriptome sequencing and analysis were performed by Anhui General Biological Co. (Anhui, China).

*1.15. Biodistribution and retention of EC NPs in vivo:*

To assess the distribution and retention of EC NPs in vivo, mice were labeled with Cy5.5 fluorescent dye and divided into groups injected with Cy5.5 alone and Cy5.5-labeled EC NPs. The distribution and retention of EC NPs in the knee joints were monitored at different time points after injection (0,3,6,9,12 and 15 days) using an in vivo imaging system (IVIS Spectrum, PerkinElmer, USA) to monitor the fluorescence intensity of Cy5.5 in mouse knee joints.

*1.16. Animals:*

In accordance with animal husbandry standards, C57BL/6 mice were housed in groups under a 12-hour light-dark cycle at 23°C to 25°C, with free access to water and standard laboratory chow.

All animal experiments were approved by the Animal Ethics Committee of Fuzhou University Affiliated Provincial Hospital (Approval Number: IACUC-FPH-SL-20250207[0507]).

*1.17. Destabilization of the medial meniscus (DMM) model of mice:*

All animal care and experimental protocols were performed by the Animal Ethics and Experimentation Committee of the Provincial Hospital of Fuzhou University. To avoid gender-dependent differences, only males were used. Eight-week-old C57BL/6 mice were selected to establish the Destabilization of the Medial Meniscus (DMM) model. Sodium pentobarbital was used for complete anesthesia. Shave the surgical area, flex the knee joint to 90°, and thoroughly disinfect. A longitudinal incision was made along the medial side of the joint, followed by careful exposure of the medial meniscus layer by layer, incision of the medial meniscus tibial ligament, and establishment of the joint instability model. The mice were divided into sham, PBS, EGCG, Cys, and EC NPs groups (n = 5). All mice received intra-articular injections of 10 µL saline or 20 μg/mL Cys, EGCG, or EC NPs every 2 weeks for 8 weeks. After 2 months postoperatively, the knee joints of the mice were removed for subsequent studies.

*1.18. Micro-CT Scanning and Analysis:*

Eight weeks after surgery, the mice were euthanized and the experimental mouse knee joints were removed intact. Samples were fixed in 4% paraformaldehyde solution for 3 days, and mouse knee specimens were analyzed using microcomputed tomography (MicroCT, Skyscan 1176, Bruker μCT, Kontich, Belgium).

*1.19. Histological staining:*

At the end of Micro-CT analysis, mouse knee samples were soaked in decalcification solution in EDTA for 14 days, dehydrated, and embedded in paraffin. The samples were cut into 3 μm thick sections using a paraffin slicer. Subsequently, they were stained with hematoxylin & eosin (HE), Safranin O-Fixed Green (SO-FG) and Toluidine Blue (TB) to analyze the structural changes of cartilage. Finally, OARSI and Mankin scores were performed on the stained sections of each group.

*1.20. Immunohistochemical (IHC) staining:*

Immunohistochemical staining was performed on the above-mentioned 4 μm mouse knee joint sections using multiple antibodies (Col-2, ACAN, MMP-13, SOX9, GPX4, ACSL4), followed by imaging using high-content screening (Opera Phenix Plus, PerkinElmer, USA).

*1.21. Biological safety evaluation:*

Mice were divided into an experimental group (administered EC NPs) and a control group. After 8 weeks of treatment, the mice were euthanized, and the major organs (heart, liver, spleen, lungs, and kidneys) were removed and immersed in 4% paraformaldehyde fixative. Tissues were paraffin-embedded and sectioned into 3-μm-thick slices. The sections were stained with hematoxylin for 3–5 minutes, washed, counterstained with eosin, dehydrated, cleared, and mounted. Finally, histological examination was performed to observe for any pathological changes.

*1.22. Statistical analysis:*

Data were analyzed using GraphPad Prism 10.0 (GraphPad software Inc., CA, USA). Data are expressed as mean ± standard deviation (SD). Statistical comparisons between two groups were performed by t-test, and statistical comparisons between three or more groups were performed by one-way ANOVA. Statistical significance was * p < 0.05, ** p < 0.01, *** p < 0.001, **** p < 0.0001.


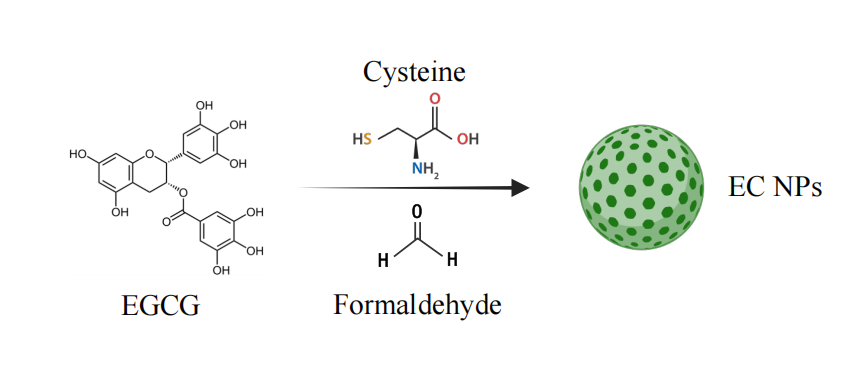


**Figure S1.** Synthesis scheme of EC NPs.


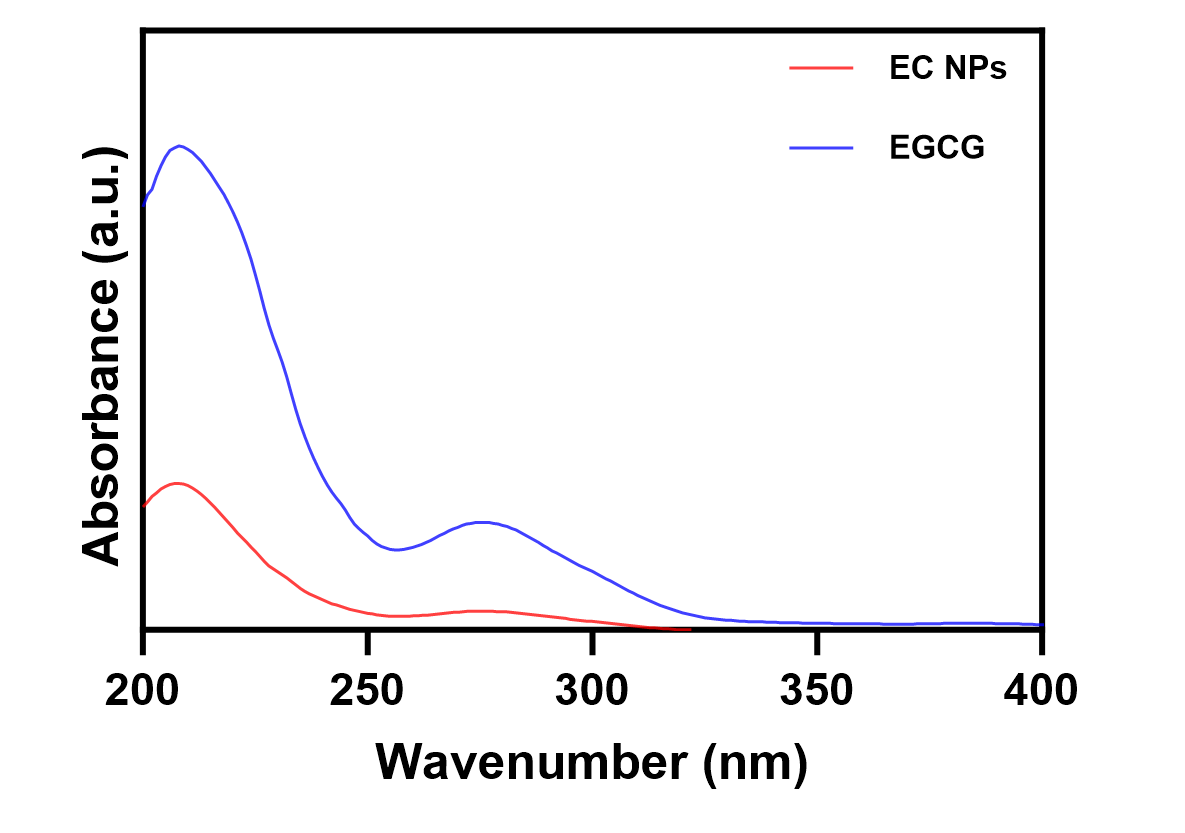


**Figure S2.** UV-visible spectra of EC NPs and EGCG.


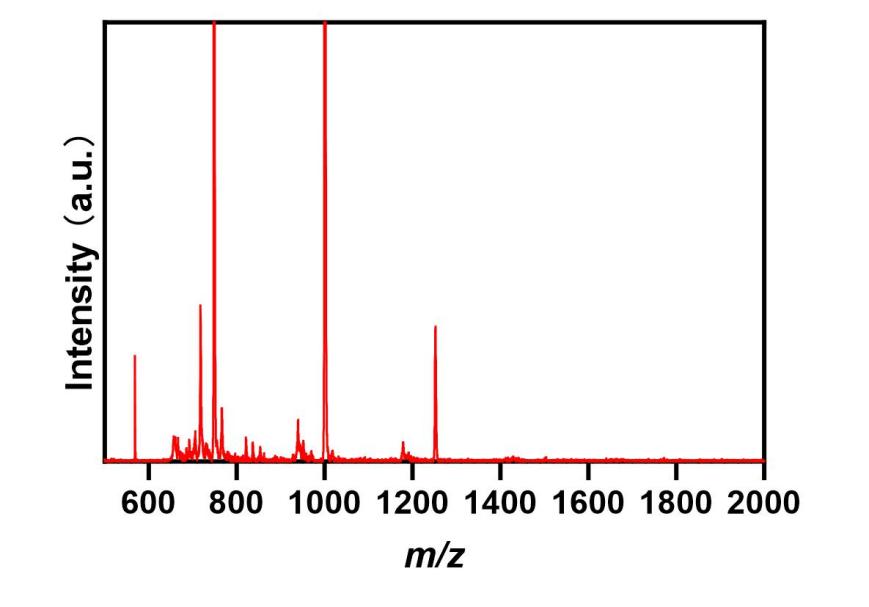


**Figure S3.** MALDI-TOF mass spectrometry of EC NPs in CHCA matrix reflection mode.


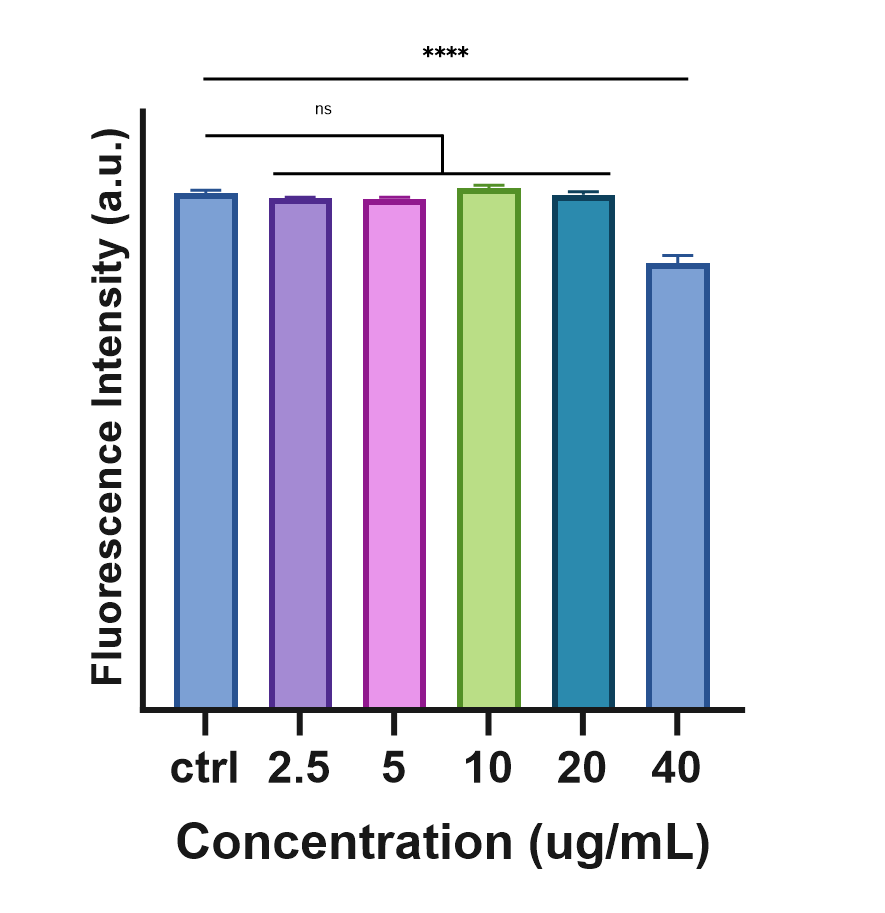


**Figure S4.** Fluorescence intensity of live-dead staining. The error bars indicate the mean ± standard deviation. ns (no statistical significance), *P < 0.05, **P < 0.01, ***P < 0.001, ****P < 0.0001.


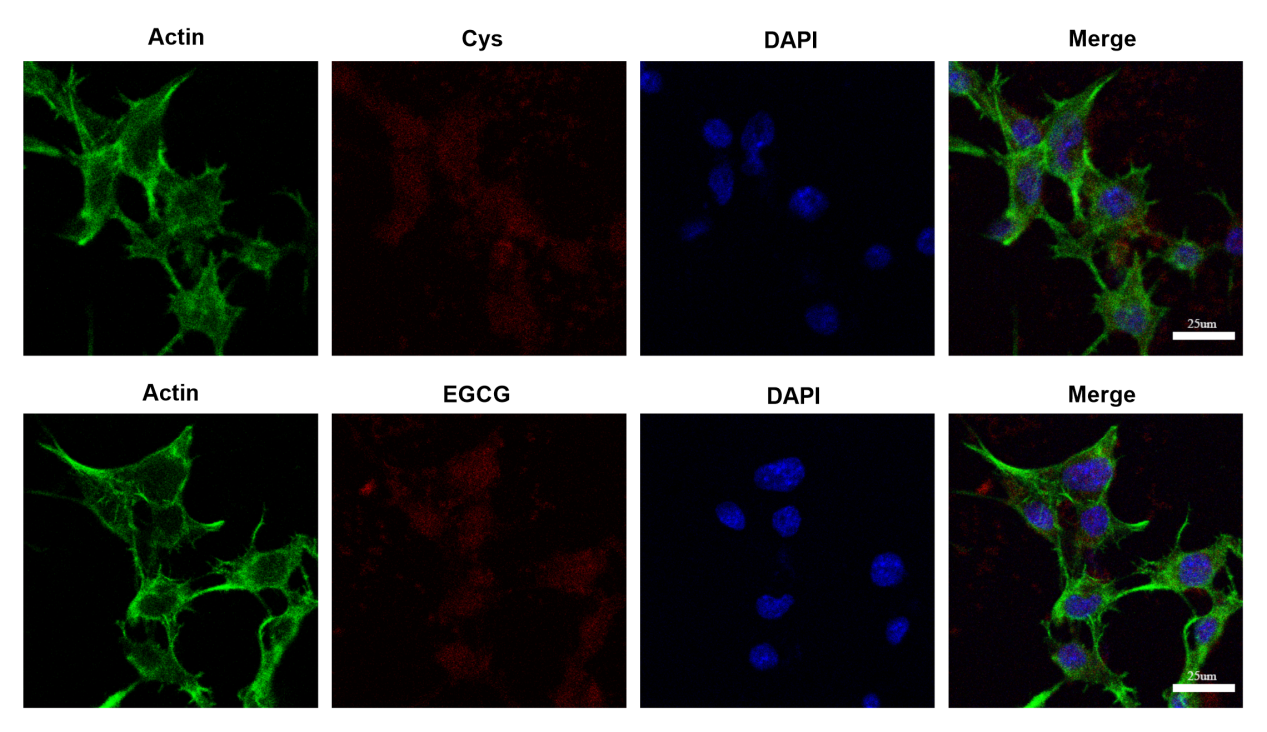


**Figure S5.** Images of chondrocyte uptake of Cys and EGCG.


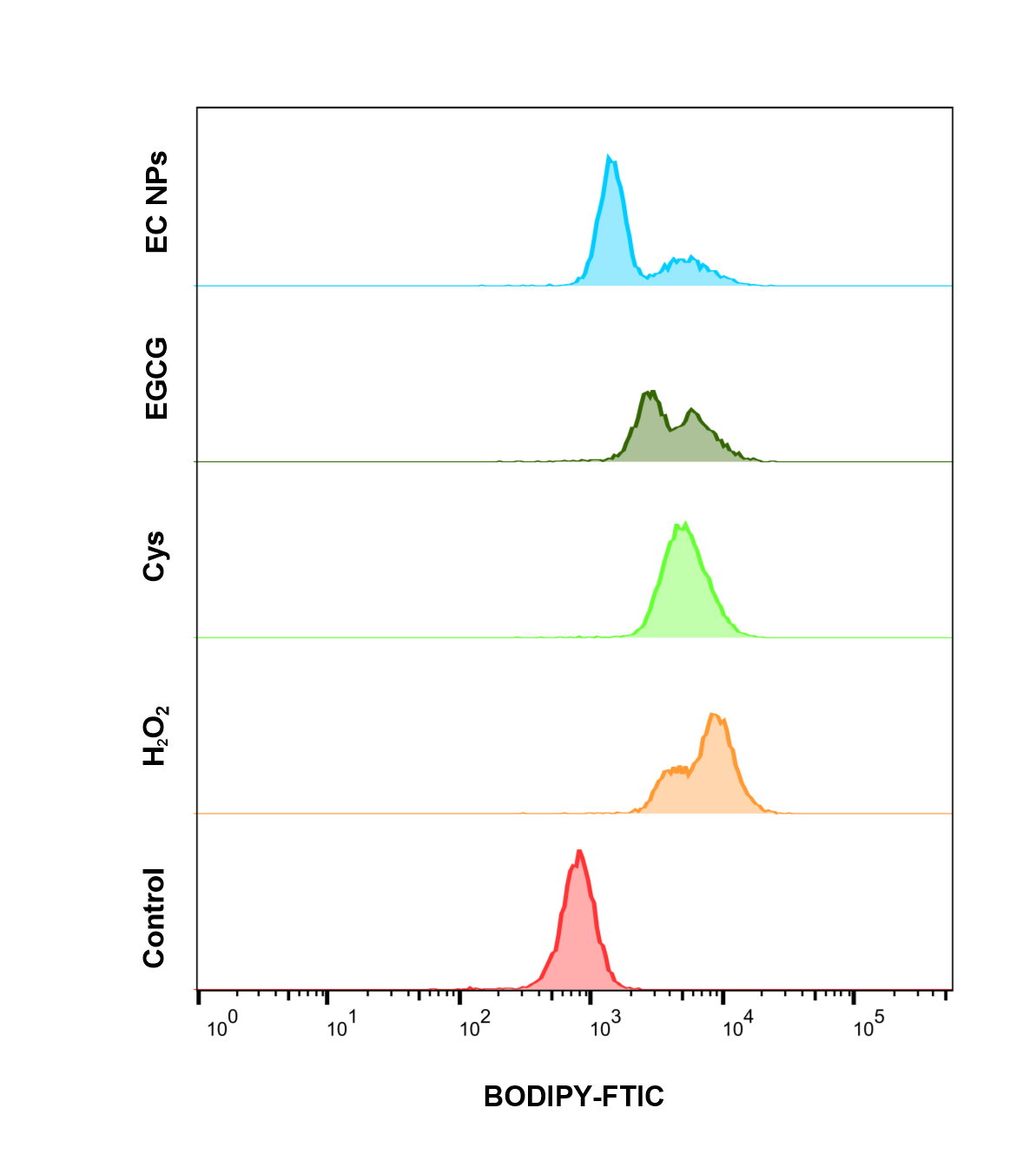

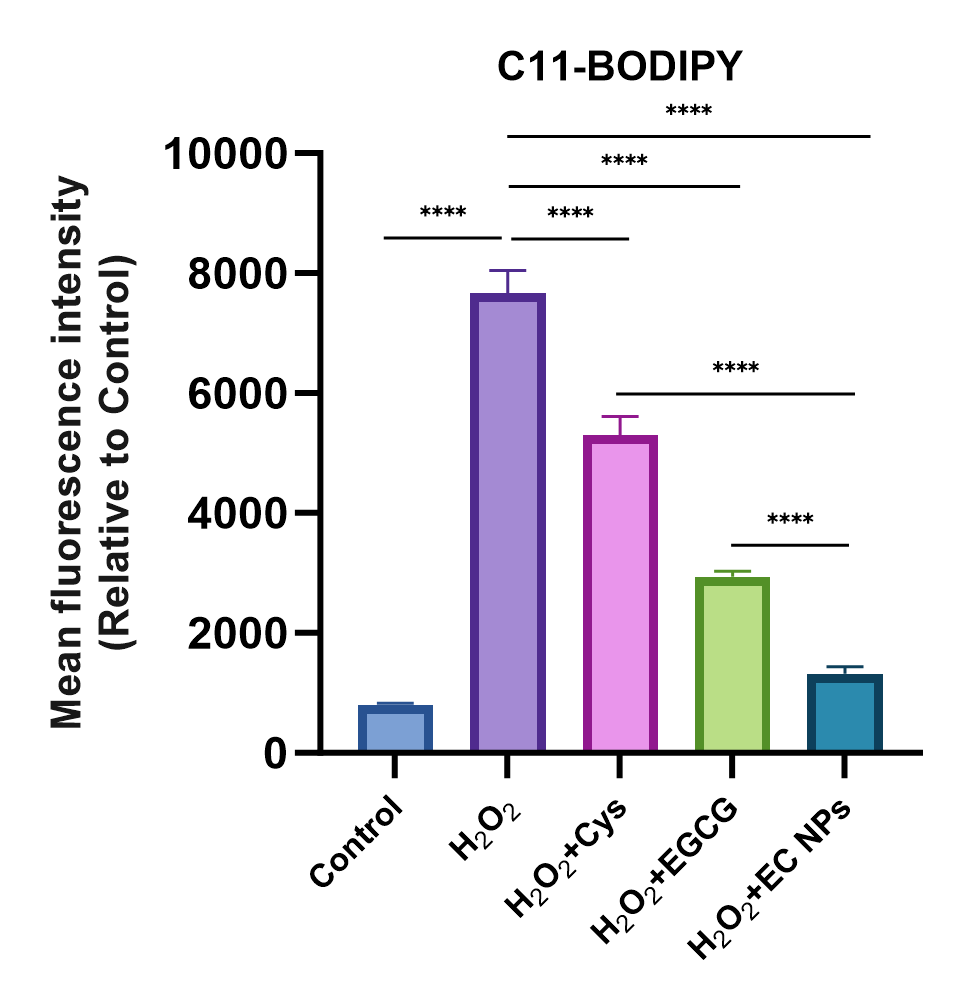


**Figure S6A Figure S6B**

**Figure S6. (A)** Flow cytometry analysis using C11-BODIPY probes measured the expression levels of lipid ROS in chondrocytes across different groups. **(B)** The expression levels of lipid ROS in chondrocytes from each group were determined using the C11-BODIPY probe. The error bars indicate the mean ± standard deviation. ns (no statistical significance), *P < 0.05, **P < 0.01, ***P < 0.001, ****P < 0.0001.


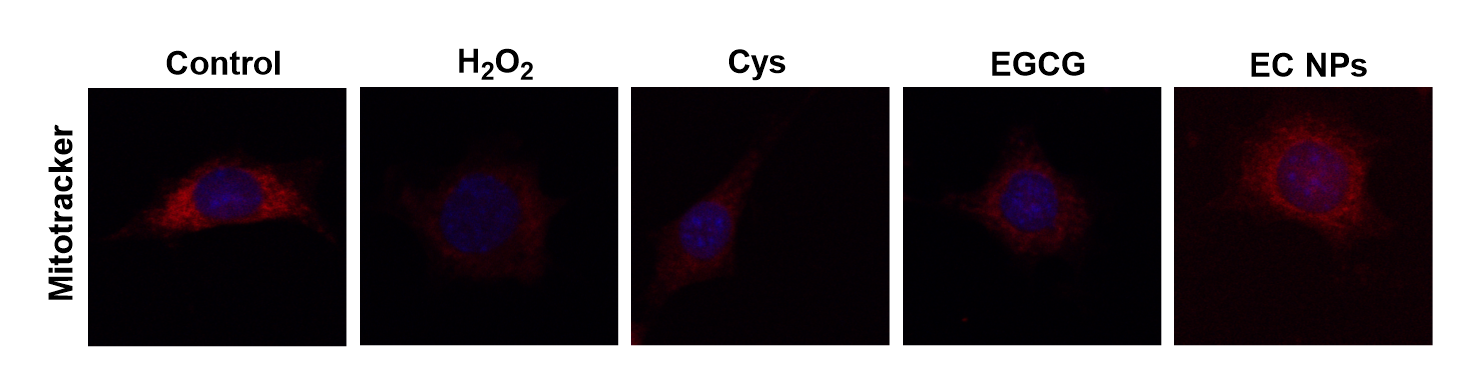


**Figure S7.** Mitotracker images of ATDC5 cells under different treatments.


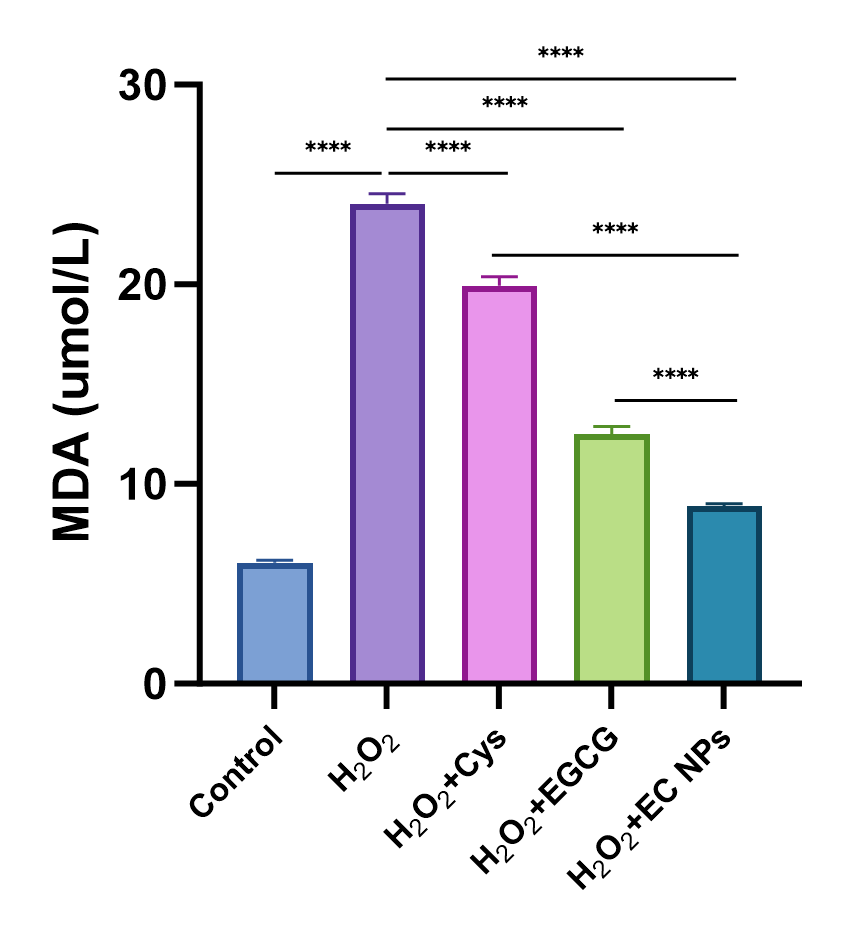


**Figure S8.** MDA levels in each group were quantified using an enzyme-linked immunosorbent assay. The error bars indicate the mean ± standard deviation. ns (no statistical significance), *P < 0.05, **P < 0.01, ***P < 0.001, ****P < 0.0001.


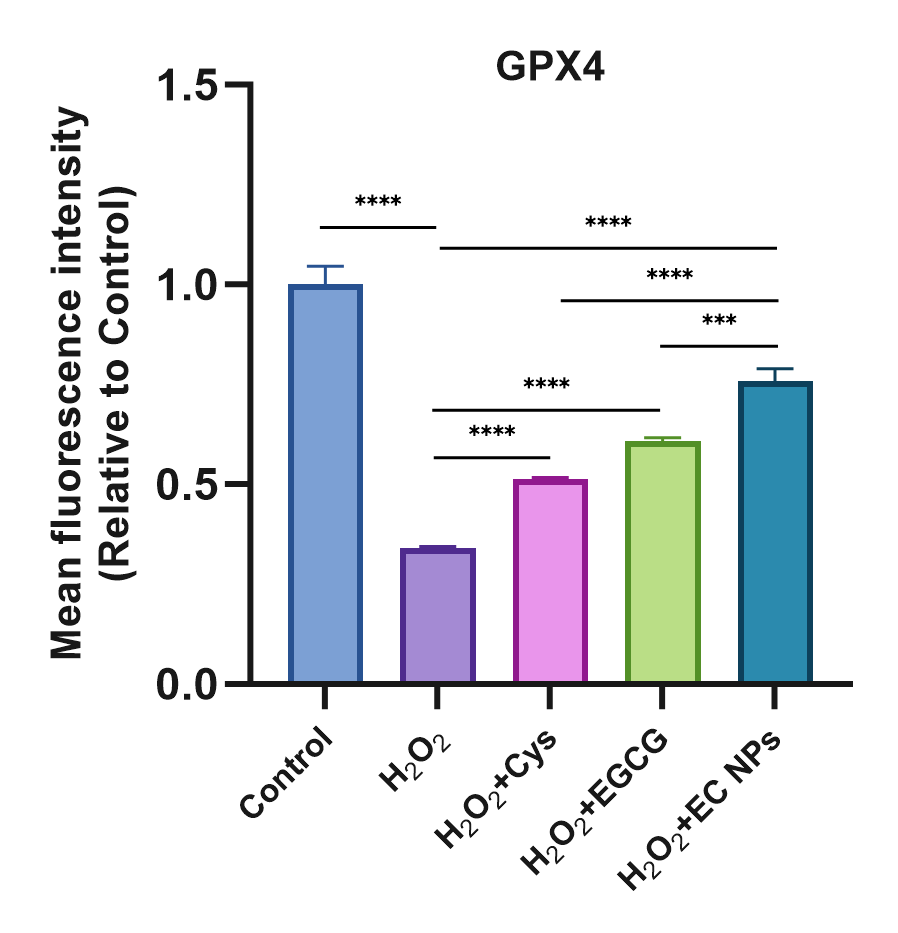


**Figure S9.** Quantitative analysis of GPX4 immunofluorescence intensity in chondrocytes from each group. The error bars indicate the mean ± standard deviation. ns (no statistical significance), *P < 0.05, **P < 0.01, ***P < 0.001, ****P < 0.0001.


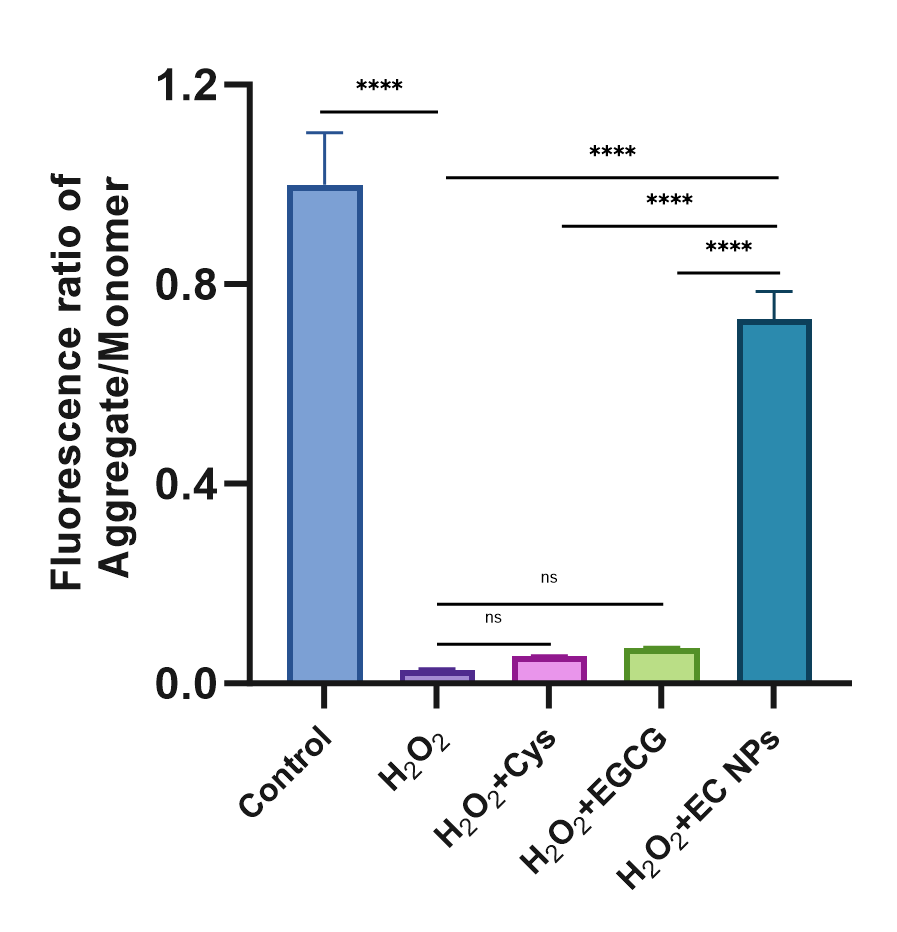


**Figure S10.** Quantitative analysis of mitochondrial membrane potential in chondrocytes from each group. The error bars indicate the mean ± standard deviation. ns (no statistical significance), *P < 0.05, **P < 0.01, ***P < 0.001, ****P < 0.0001.


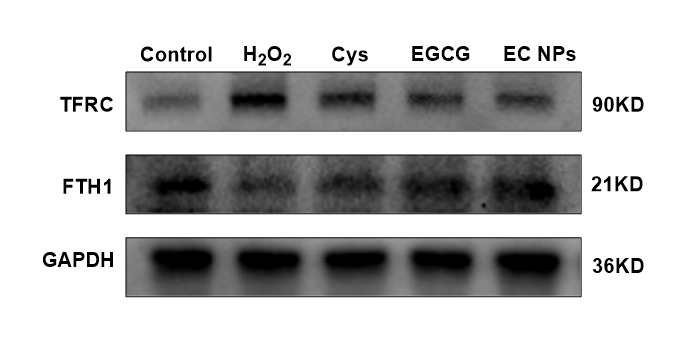
**
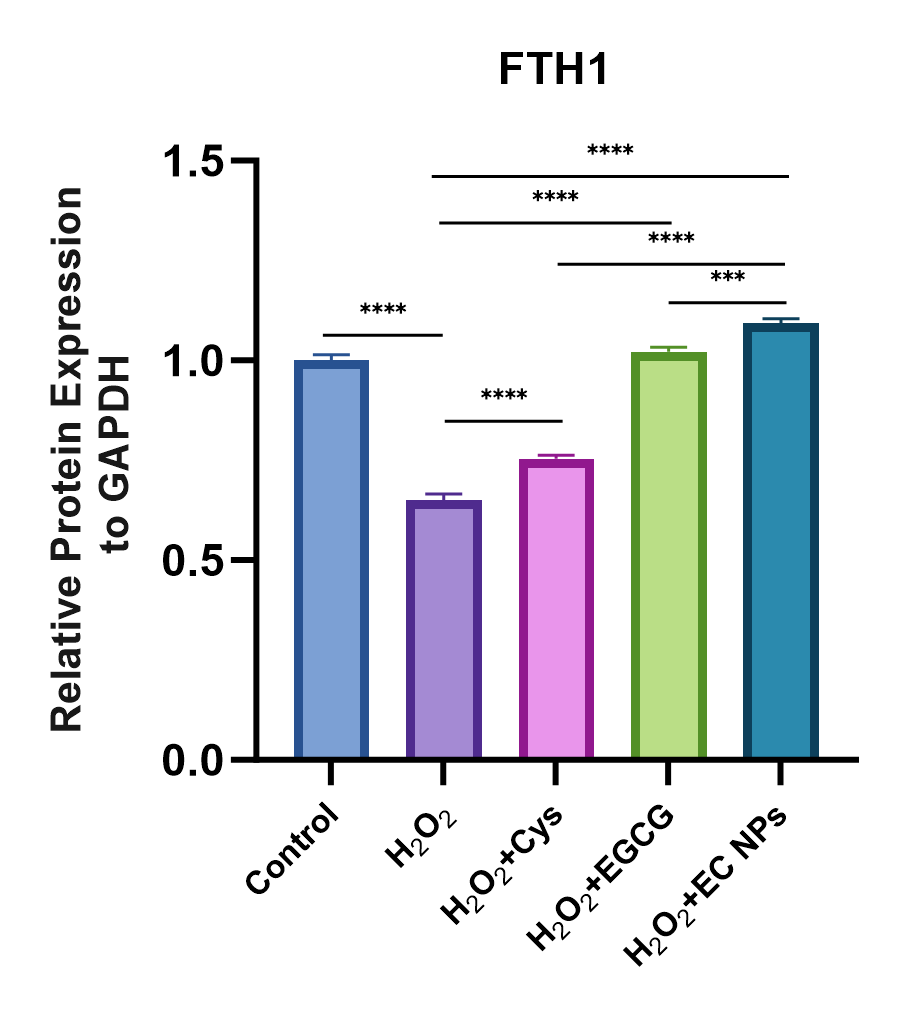
**
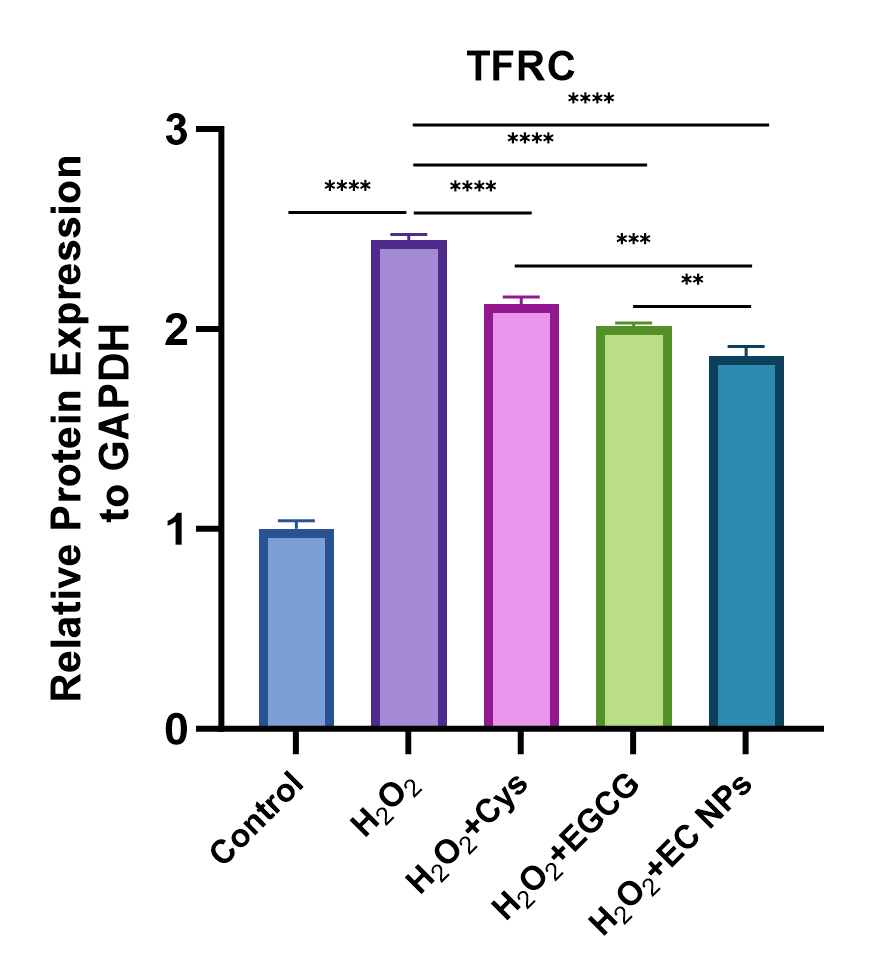


**Figure S11A Figure S11B Figure S11C**

**Figure S11. (A)** Western blot for FTH1 and TFRC protein expression. **(B,C)** Relative expression levels of FTH1 and TFRC protein. The error bars indicate the mean ± standard deviation. ns (no statistical significance), *P < 0.05, **P < 0.01, ***P < 0.001, ****P < 0.0001.


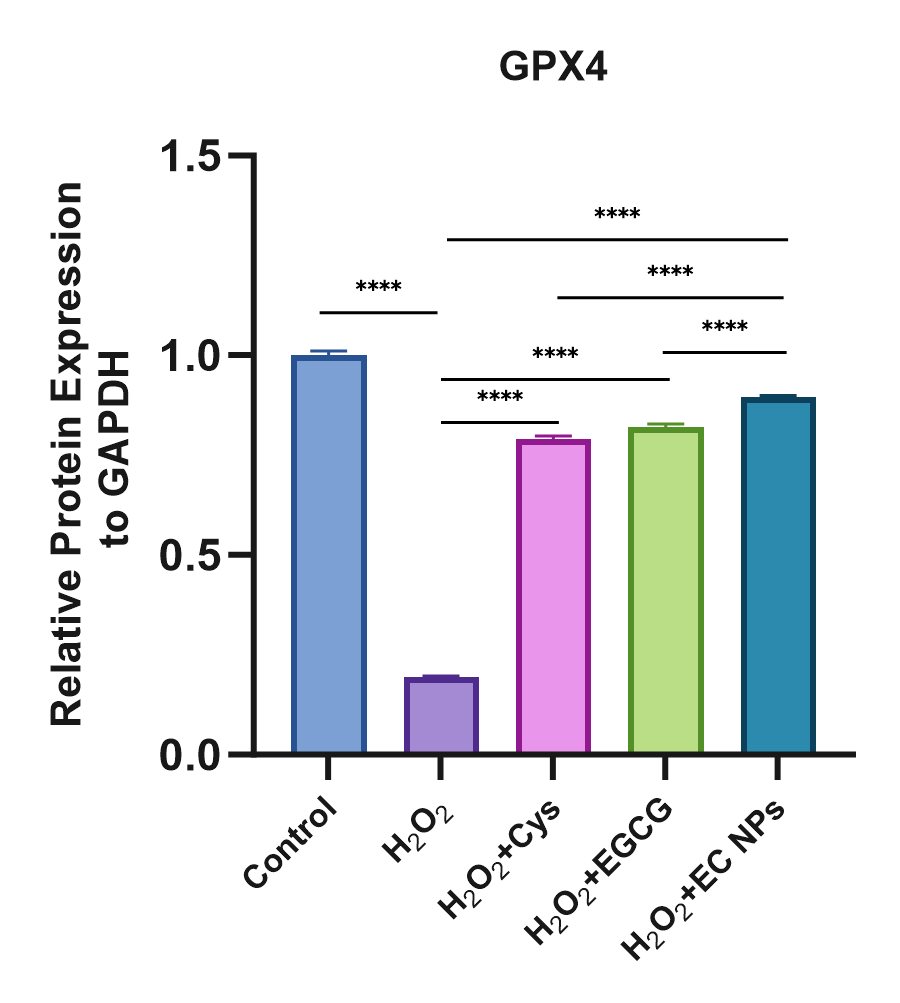

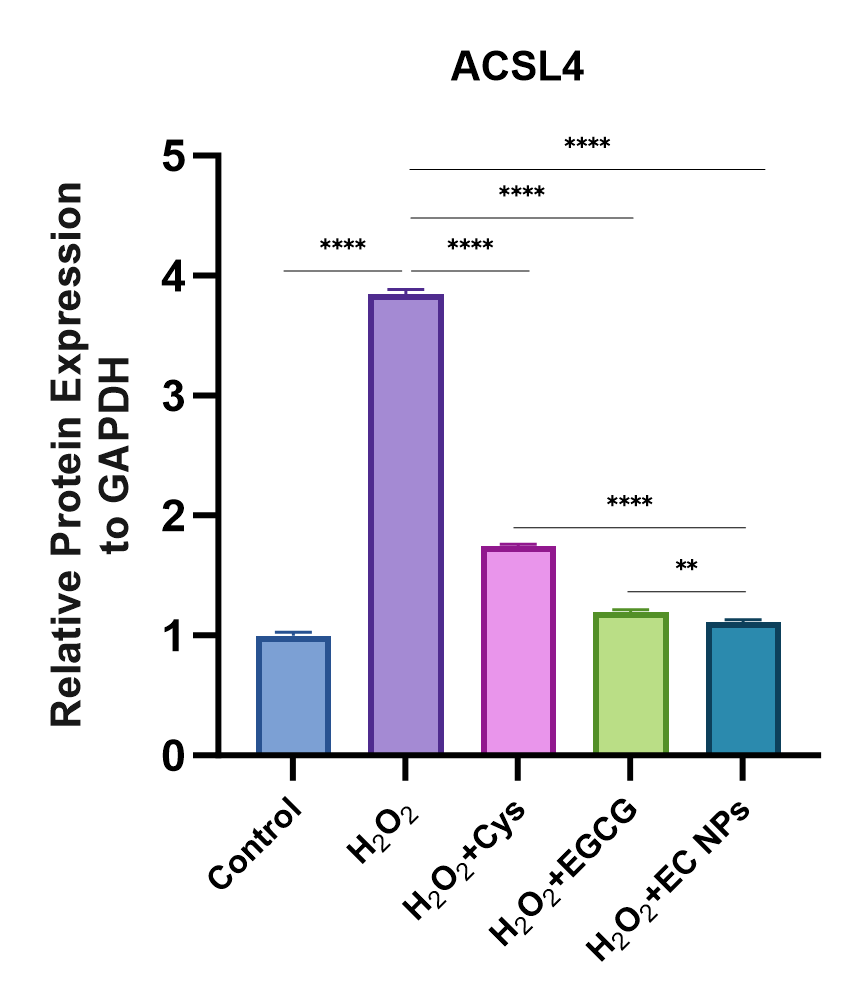


**Figure S12.** Relative expression levels of GPX4 and ACSL4 protein. The error bars indicate the mean ± standard deviation. ns (no statistical significance), *P < 0.05, **P < 0.01, ***P < 0.001, ****P < 0.0001.


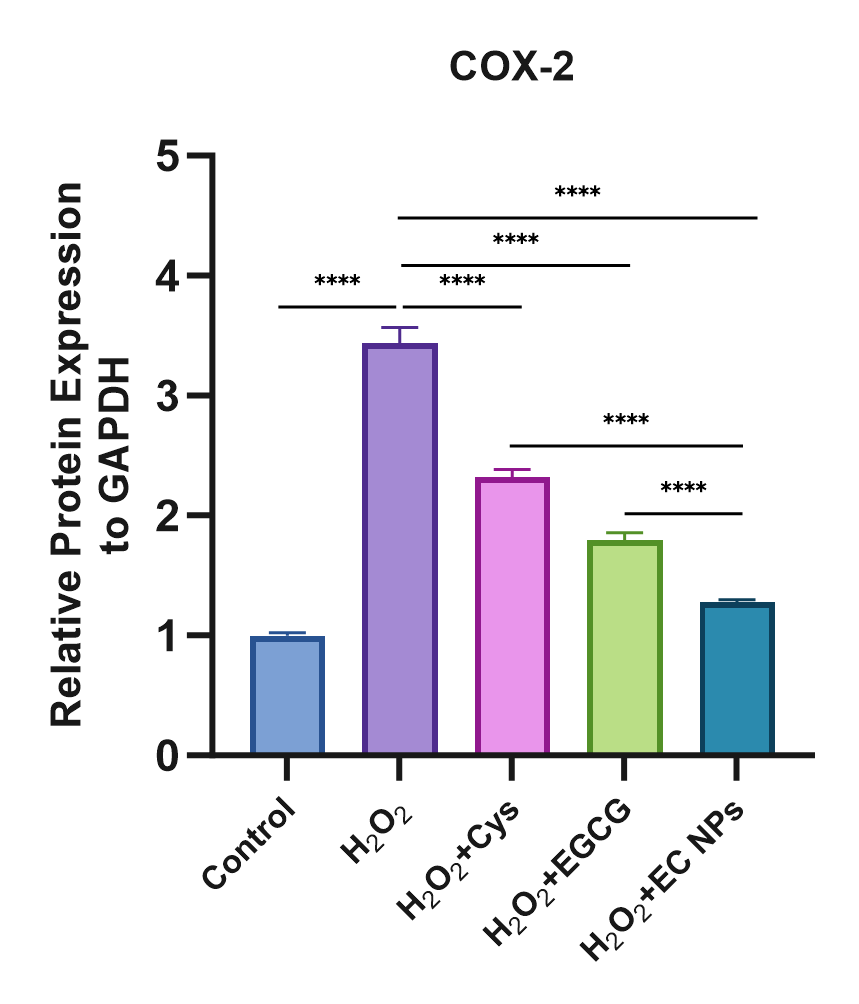

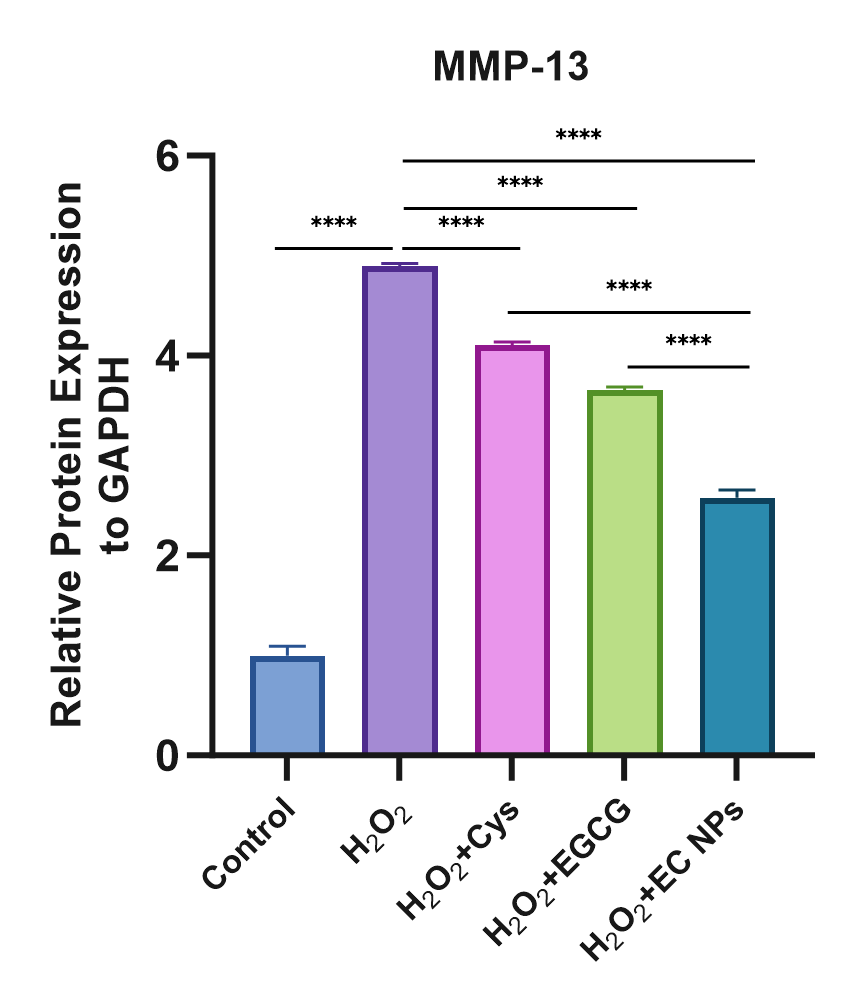

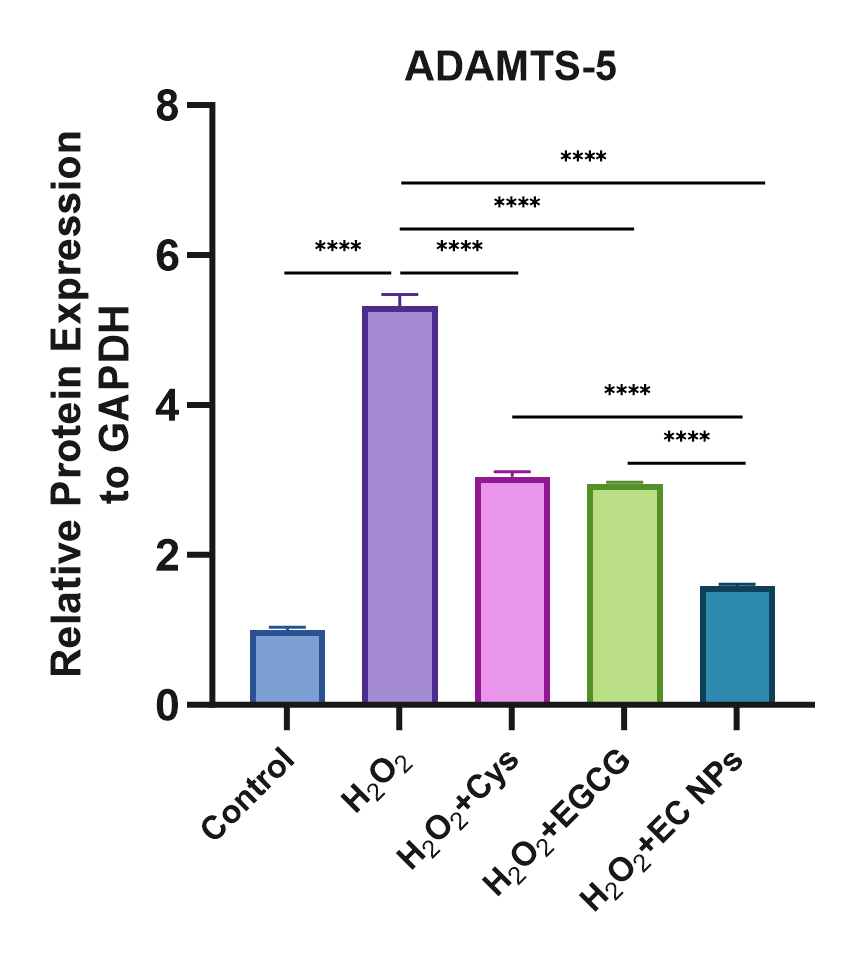


**Figure S13.** Relative protein expression levels of COX-2, MMP-13, and ADAMTS-5. The error bars indicate the mean ± standard deviation. ns (no statistical significance), *P < 0.05, **P < 0.01, ***P < 0.001, ****P < 0.0001.


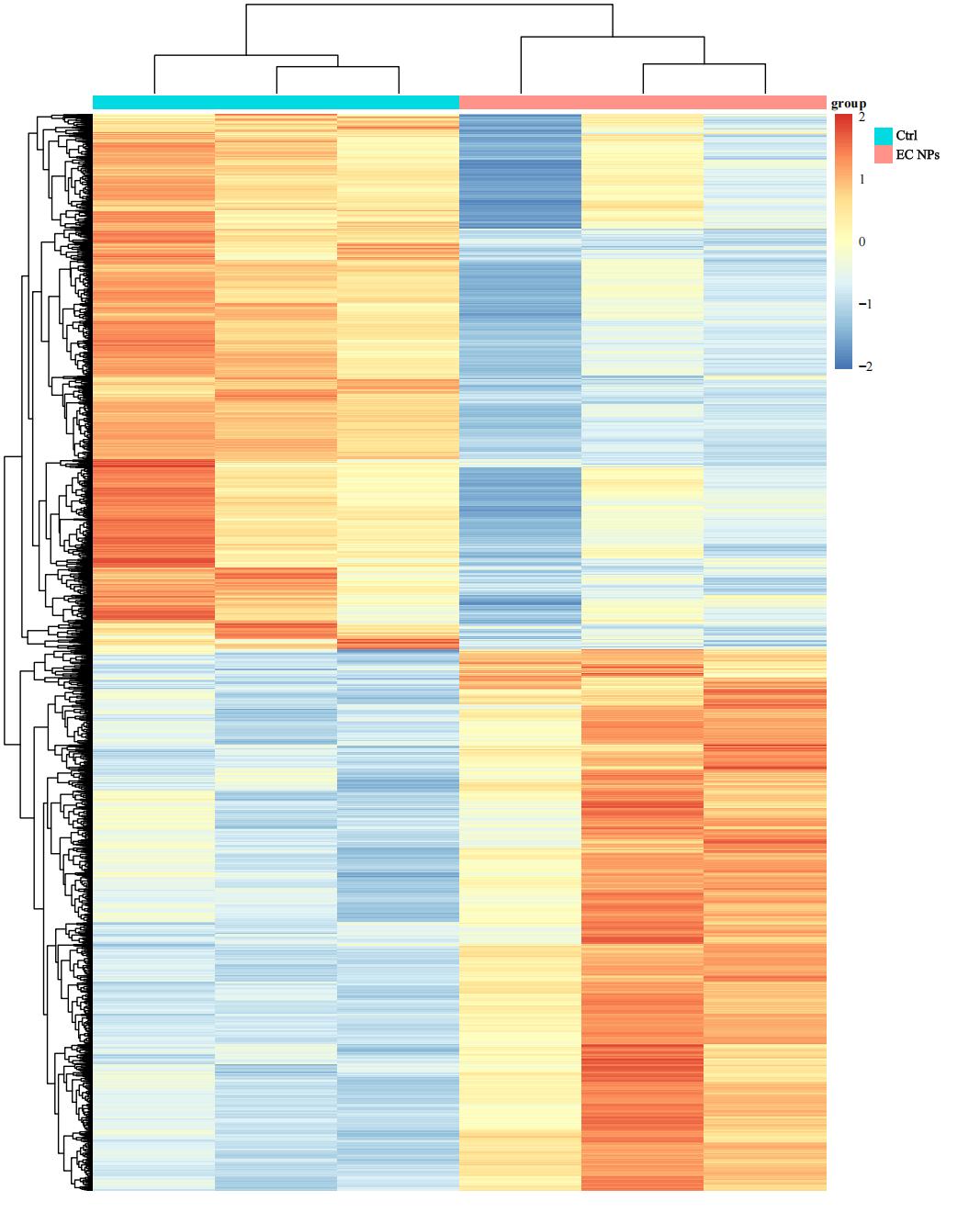


**Figure S14.** Heatmap of differential genes in H_2_O_2_-stimulated control and EC NPs groups.


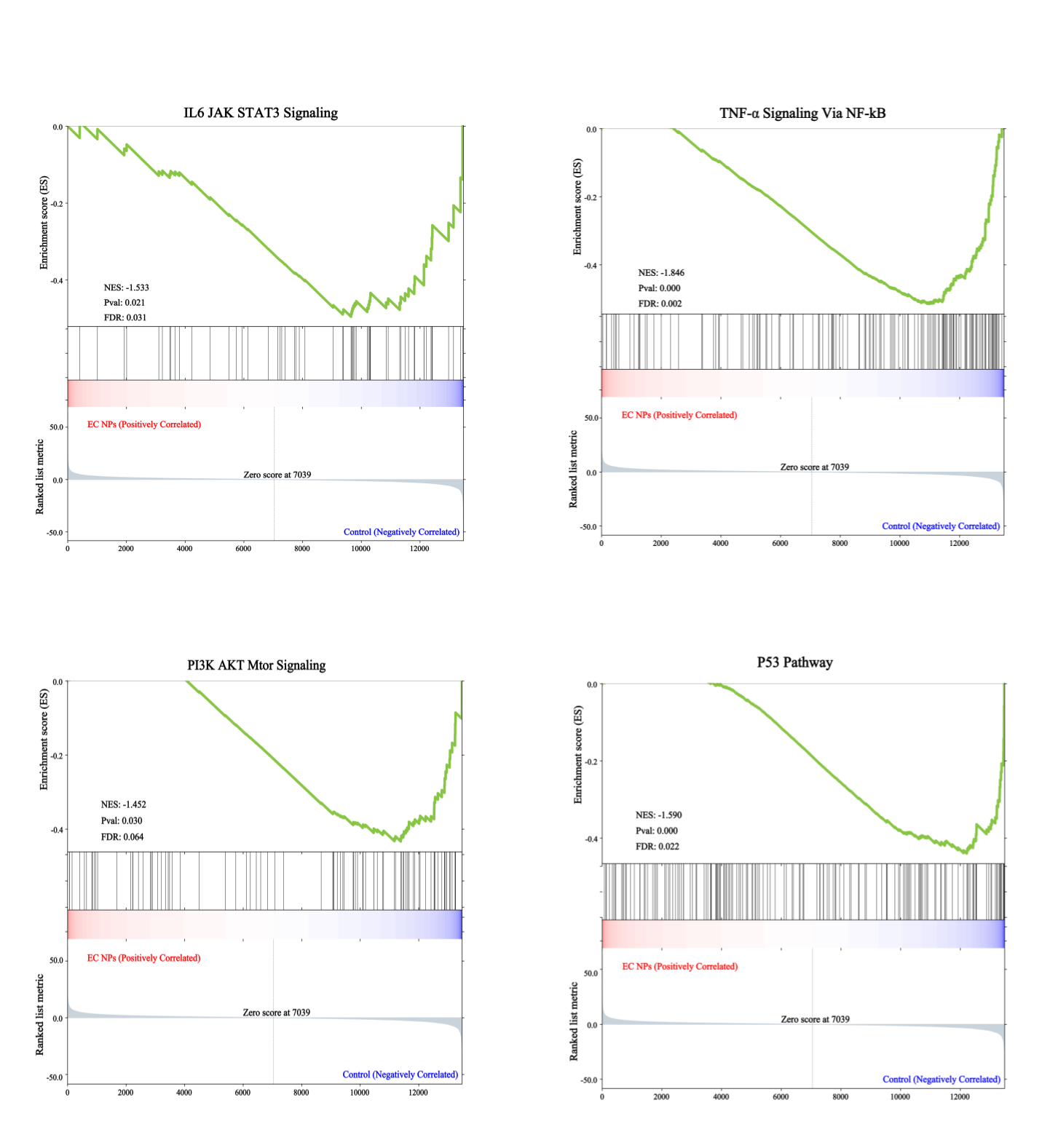


**Figure S15.** IL6 JAK STAT3 signaling, TNF-α signaling via NF-κB, PI3K AKT Mtor signaling, and P53 pathway based on GSEA analysis


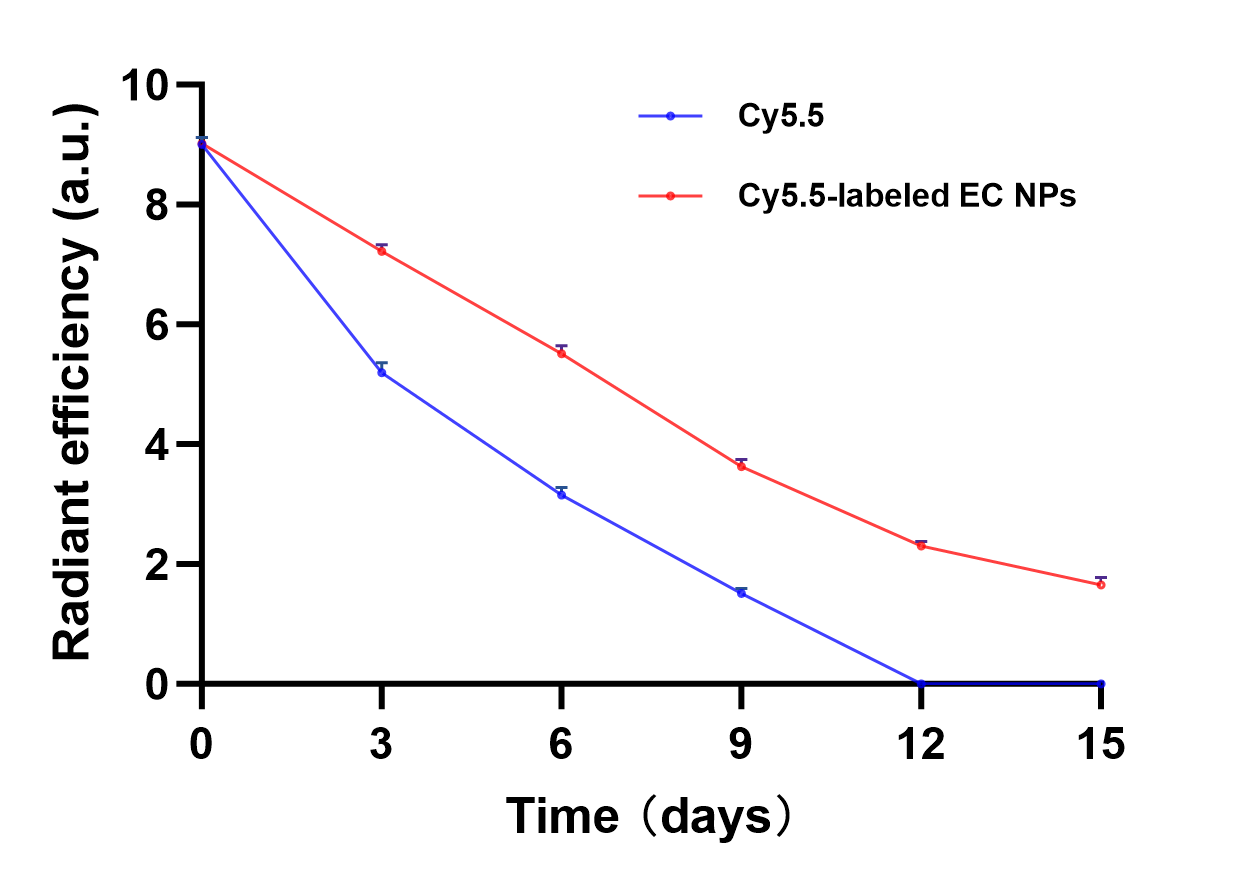


**Figure S16.** Fluorescence intensity of Cy5.5 dye and Cy5.5-labeled EC NPs injected into the knee joint.


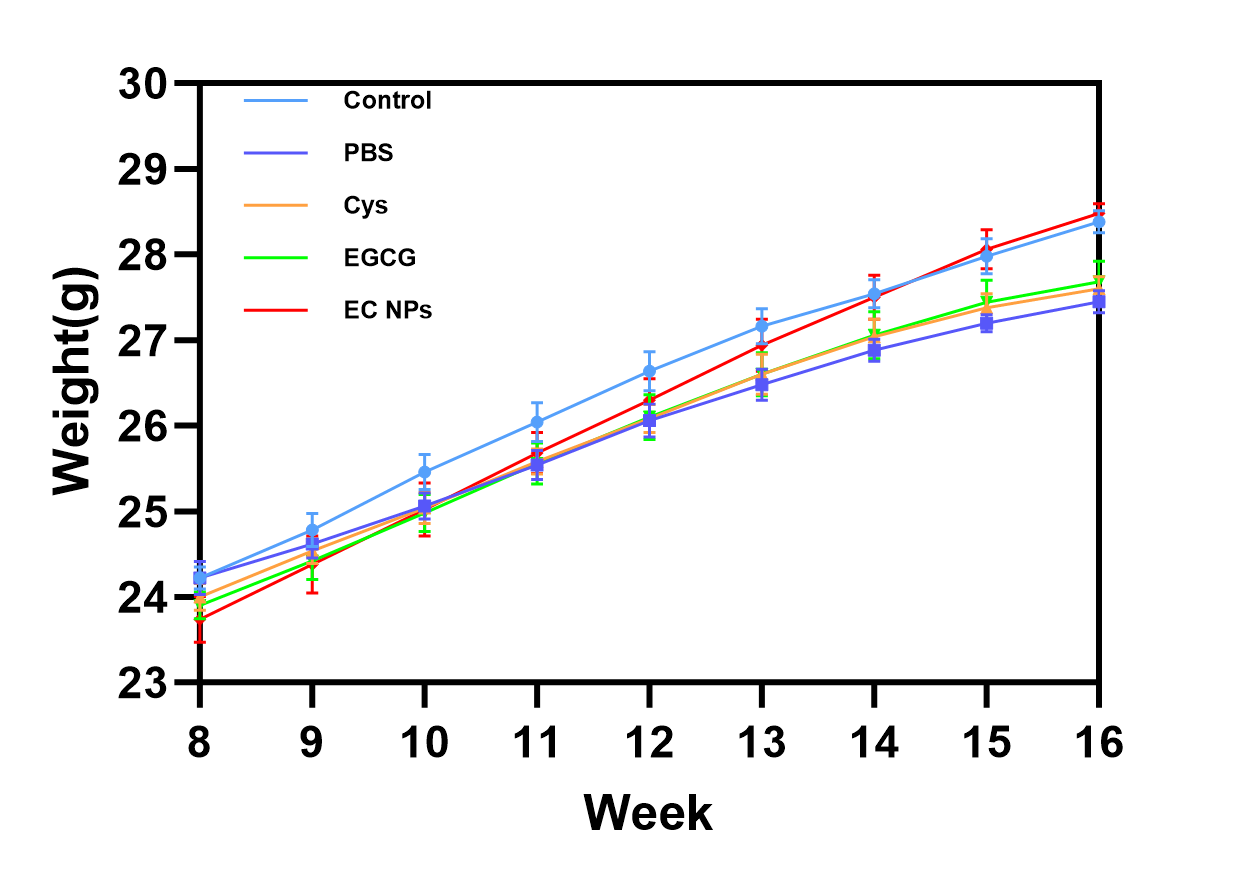


**Figure S17.** Weight changes over time in each group of mice.


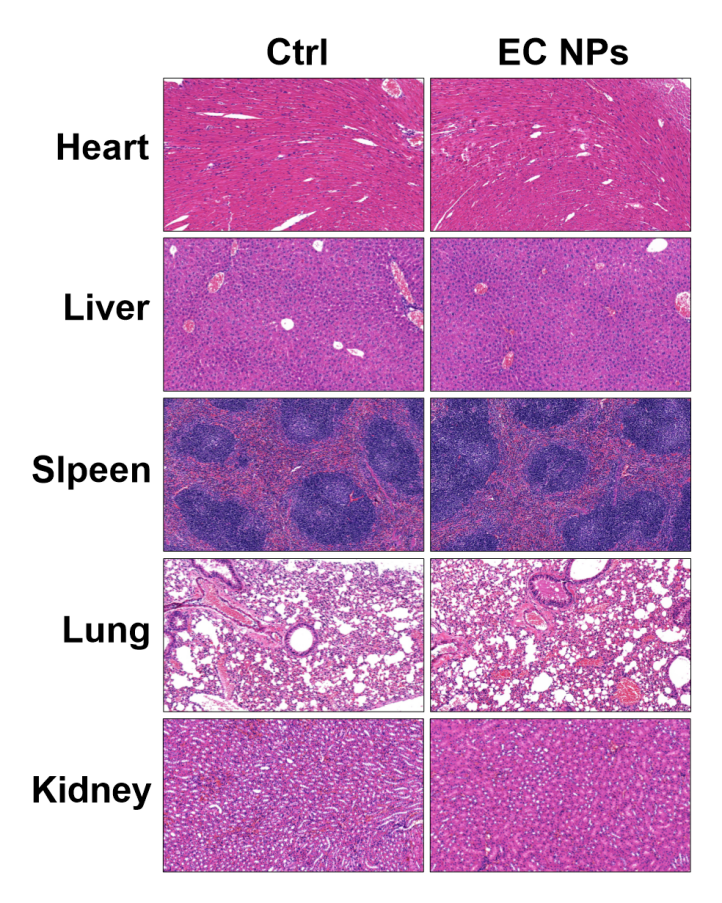


**Figure S18.** The hearts, livers, spleens, lungs, and kidneys of mice in the control group and EC NPs group were subjected to HE staining.

**Table S1. Primer sequences for genes used in RT-qPCR experiments.**

| **Primer** | **Primer sequence** | **product size** |
| --- | --- | --- |
| GAPDH-F | GGTGAAGGTCGGTGTGAACG | 20 |
| GAPDH-R | CTCGCTCCTGGAAGATGGTG | 20 |
| GPX4-F | GATGGAGCCCATTCCTGAACC | 21 |
| GPX4-R | CCCTGTACTTATCCAGGCAGA | 21 |
| ACSL4-F | ATTGGTCAGGGATATGGGCT | 20 |
| ACSL4-R | AGAGGAGCTCCAACTCTTCCA | 21 |
| COL2A1-F | TGGTCCTCTGGGCATCTCAGGC | 22 |
| COL2A1-R | GGTGAACCTGCTGTTGCCCTCA | 22 |
| MMP13-F | TTTGAGAACACGGGGAAGA | 20 |
| MMP13-R | ACTTTGTTGCCAATTCCAGG | 20 |
| ADAMTS5-F | GGAGCGAGGCCATTTACAAC | 20 |
| ADAMTS5-R | CGTAGACAAGGTAGCCCACTTT | 22 |
| ACAN-F | GTGGAGCCGTGTTTCCAAG | 19 |
| ACAN-R | AGATGCTGTTGACTCGAACCT | 21 |
| SOX9-F | CACAAGAAAGACCACCCCGA | 20 |
| SOX9-R | CTCCGCTTGTCCGTTCTTCA | 20 |
| COX-2-F | GCATTCTTTGCCCAGCACTT | 20 |
| COX-2-R | ACCTCTCCACCAATGACCTGA | 21 |
| iNOS-F | CCTGCTTTGTGCGAAGTGTC | 20 |
| iNOS-R | CCCAAACACCAAGCTCATGC | 20 |
